# Diel-driven variations of leaf dark respiration and metabolite levels in C_3_ and C_4_ grasses

**DOI:** 10.1093/plphys/kiag200

**Published:** 2026-04-13

**Authors:** Yuzhen Fan, Guillaume Tcherkez, Andrew P Scafaro, Nicolas L Taylor, Robert T Furbank, Susanne von Caemmerer, Owen K Atkin

**Affiliations:** ARC Centre of Excellence in Plant Energy Biology, Research School of Biology, The Australian National University, Canberra, ACT, Australia; Division of Plant Sciences, Research School of Biology, The Australian National University, Canberra, ACT, Australia; Division of Plant Sciences, Research School of Biology, The Australian National University, Canberra, ACT, Australia; Institut de Recherche en Horticulture et Semences, INRAE, Université d'Angers, Beaucouzé, France; ARC Centre of Excellence in Plant Energy Biology, Research School of Biology, The Australian National University, Canberra, ACT, Australia; Division of Plant Sciences, Research School of Biology, The Australian National University, Canberra, ACT, Australia; School of Molecular Sciences, Australian Plant Phenomics Network, ARC Training Centre in Predictive Breeding for Agricultural Futures and The UWA Institute of Agriculture, The University of Western Australia, 35 Stirling Highway, Crawley, Perth, WA 6009, Australia; Division of Plant Sciences, Research School of Biology, The Australian National University, Canberra, ACT, Australia; ARC Centre of Excellence for Translational Photosynthesis, Research School of Biology, The Australian National University, Canberra, ACT, Australia; Division of Plant Sciences, Research School of Biology, The Australian National University, Canberra, ACT, Australia; ARC Centre of Excellence for Translational Photosynthesis, Research School of Biology, The Australian National University, Canberra, ACT, Australia; ARC Centre of Excellence in Plant Energy Biology, Research School of Biology, The Australian National University, Canberra, ACT, Australia; Division of Plant Sciences, Research School of Biology, The Australian National University, Canberra, ACT, Australia

## Abstract

Leaf dark respiration (*R*_dark_) is often measured in artificially dark-adapted samples at a single time point during the day, with temperature-normalized rates often assumed to be constant throughout a 24-h cycle. However, the extent to which the 24-h cycle influences leaf *R*_dark_ and respiratory metabolic profile remains unclear, particularly in C_4_ plants. We quantified O_2_-based leaf *R*_dark_ and metabolites at 6 time points over a diel (ie 24-h) cycle in leaves of 4 grass species (1 C_3_ and 3 C_4_ species). We found that *R*_dark_ and leaf metabolites varied among species and between dark-adapted day-sampled and night-sampled leaves. In general, C_4_ plants contained a relatively higher content of organic acids and soluble sugars than C_3_ plants. Across the 4 species, variations in *R*_dark_ were associated with changes in the abundance of metabolites involved in the mitochondrial tricarboxylic acid pathway (malate, fumarate, succinate, and citrate), amino acid metabolism (alanine and asparagine), and sugar interconversion (lactose and mannose). Multivariate statistics suggested that *R*_dark_ of the examined species is influenced more by the relative contribution of multiple concurrent metabolic pathways across the diel cycle than by C_3_ and C_4_ photosynthetic types. We suggest that *R*_dark_ and metabolite profiles measured during daytime on dark-adapted leaves are not good surrogates for nighttime respiratory properties. Understanding how the supply of respiratory substrates varies during the diel cycle would lead to a more accurate prediction of *R*_dark_ over the course of a day.

## Introduction

Respiration is a crucial process for plant primary production, as it provides energy and carbon skeletons necessary for growth and maintenance. Past studies have shown that leaf respiration rates are dynamic, varying with growth and measuring conditions (Wright et al. 2006; Atkin et al. 2015), genotypes (Scafaro et al. 2017; Bulut et al. 2023; Gaju et al. 2025), and the diel cycle (Way et al. 2015; Rashid et al. 2020; Bruhn et al. 2022). Variations in steady-state leaf respiration rate in darkness (*R*_dark_) can reflect variations in ATP demand (eg to support protein turnover and ion gradient generation), with respiratory rates scaling with leaf nitrogen ([N]) content in several studies. Rates of *R*_dark_ can also be influenced by the abundance and type of available substrates. For example, *R*_dark_ (measured at a single time point of the day) strongly correlates with the availability of carbohydrates and organic and amino acids in C_3_ leaves, and rates of *R*_dark_ can be simulated by adding exogenous substrates in C_3_ and C_4_ leaves (Azcón-Bieto et al. 1983; Agostino et al. 1996; Noguchi et al. 2005; Zell et al. 2010; O’Leary et al. 2017; Fan et al. 2022b). Other studies revealed that variation in nocturnal leaf *R*_dark_ of C_3_ plants is mainly underpinned by changes in the pool sizes and types of respiratory substrate used rather than by environmental factors such as temperature (Tcherkez et al. 2003; Bruhn et al. 2022, 2024a; Bruhn 2023; Jones et al. 2024). This suggests that the diel cycle may also affect leaf *R*_dark_ via changes in substrate availability and, accordingly, alterations in metabolic pathways.

There is evidence that rates of leaf *R*_dark_ in C_3_ plants vary throughout a day in relation to substrate availability. For example, rates of *R*_dark_ varied over a 24-h diel cycle regardless of the growth temperature in C_3_ rice leaves (Rashid et al. 2020). This diel-driven variation in *R*_dark_ was linked to changes in the availability of metabolites associated with respiratory pathways, particularly amino and organic acid intermediates of the mitochondrial tricarboxylic acid pathway (TCAP). Sugar concentration is often positively correlated with diurnal rates of *R*_dark_ in C_3_ leaves (McCree 1974; Azcón-Bieto and Osmond 1983; Noguchi et al. 1996; Ögren 2000; Griffin et al. 2002; Whitehead et al. 2004). In C_3_ Arabidopsis leaf discs, rates of *R*_dark_ vary in response to exogenous application of carbohydrates and organic and amino acids (O’Leary et al. 2017). In addition, high concentrations of amino acids can activate the rapamycin kinase signaling pathway, downregulating mitochondrial activity and associated rates of *R*_dark_ in Arabidopsis (Mallén-Ponce et al. 2022). These results show that leaf *R*_dark_ in C_3_ plants can fluctuate over the diel cycle, and these fluctuations may be associated with changes in multiple metabolites and pathways.

In contrast to our growing understanding of how leaf *R*_dark_ varies over time in C_3_ plants, less is known about drivers of *R*_dark_ in C_4_ plants. C_4_ plants use a specialized CO_2_-concentrating mechanism to increase the CO_2_ concentration around Rubisco, thereby increasing photosynthetic efficiency (von Caemmerer and Furbank 2016). This mechanism can be further categorized into 3 biochemically distinct classical types—NADP-dependent malic enzyme (NADP-ME), NAD-dependent malic enzyme (NAD-ME), and phosphoenolpyruvate carboxykinase (PCK) types, based on the decarboxylases involved in the mechanism (von Caemmerer and Furbank 2016). Each C_4_ type uses mitochondria differently in its CO_2_-concentrating mechanism (see a review by Hatch (1987)). Recently, we explored whether the different roles of mitochondria in the C_4_ mechanism could have led to distinct demands for respiratory products, potentially giving rise to variation in C_4_ leaf *R*_dark_ (Fan et al. 2022a). For example, C_4_ plants may have lower demands for respiratory energy associated with protein turnover at night, as a result of having a lower Rubisco concentration than C_3_ plants (Fan et al. 2022a). However, the rate of CO_2_-based leaf *R*_dark_ in the 3 C_4_ biochemical types is significantly higher when measured at midday compared to selected C_3_ species, and this difference was maintained at midnight (Fan et al. 2024). An additional factor that may influence differences in the rate of leaf *R*_dark_ is the type of substrates used to fuel respiratory metabolism, both between C_3_ and C_4_ plants in general, and among the 3 C_4_ biochemical types. For instance, while C_3_ plants typically rely on carbohydrates as the primary respiratory substrate, some, but not all, C_4_ plants can metabolize organic acids at high rates to fuel *R*_dark_ (Fan et al. 2022b, 2024). Understanding how organic acid content or, more generally, metabolite profiles vary across C_3_ and C_4_ species, and how they change over a day, is essential for interpreting diel variation in leaf *R*_dark_.

There has been limited information on how metabolic profiles vary over a day in C_4_ leaves. A comparison between C_3_ and C_4_ NADP-ME-type *Flaveria* species found that in vivo metabolite pools of malate, aspartate, pyruvate, and 2-oxoglutarate were on average 2-fold higher in C_4_ leaves than their C_3_ counterparts (Borghi et al. 2022; Tang et al. 2024). Similarly, the abundance of photosynthetic C_4_ acids, including malate and aspartate, has been reported to remain high throughout the night in a range of C_4_ species (Hatch 1979; Stitt and Heldt 1985; Du et al. 2000; Czedik-Eysenberg et al. 2016). As for carbohydrates, evidence suggests that leaves of C_4_ NADP-ME-type species, such as maize and sugarcane, can retain 20% to 50% of their starch content by dawn, compared to 0% to 5% in C_3_ leaves (Pilkington et al. 2015; Czedik-Eysenberg et al. 2016; De Souza et al. 2018). Presumably, elevated levels of starch (thus starch-derived carbohydrates and organic acids) in C_4_ NADP-ME leaves could potentially alter leaf *R*_dark_ rates, provided that respiratory demand is not limiting. This effect may be more pronounced in C_4_ NAD-ME and PCK types, which can remobilize organic acids, in addition to carbohydrates, to fuel *R*_dark_ (Fan et al. 2022b). To what extent changes in metabolite pools are reflected in rates of leaf *R*_dark_ remains unclear and requires further investigation.

Our recent work explored the day-night variation in leaf *R*_dark_ and targeted metabolite profiles in 2 species of C_3_ grasses and 6 species of C_4_ grasses through comparisons at midday and midnight (Fan et al. 2024). We found that in most C_4_ species examined, changes in organic acid content (particularly malate and succinate) were strongly correlated with variation in *R*_dark_, highlighting the key role of TCAP intermediates in influencing *R*_dark_ in C_4_ plants. However, it remains unclear whether the patterns observed at midday and midnight hold across the full diel cycle. Measurements with higher time resolution are required to capture the full range of changes in leaf *R*_dark_ and metabolite profiles over 24 h. Moreover, capturing a more complete metabolic profile over time is essential to evaluate whether *R*_dark_ values measured during the day using dark-adapted leaves accurately reflect nighttime respiratory metabolism.

In this current study, we conducted a detailed experiment investigating whether dark respiratory metabolism is affected by the diel cycle in leaves of one C_3_ (*Triticum aestivum*) and 3 C_4_ grass species (each representing a C_4_ biochemical type). We measure leaf *R*_dark_ (ie O_2_ uptake in darkness) and analyze dark-adapted metabolite profiles at 6 time points during the day and at night over a 24-h diel cycle. We tested the following hypotheses:

1. Rates of leaf *R*_dark_ vary over a diel cycle in a manner reflecting changes in the abundance of respiratory substrates such as carbohydrates and organic acids.
2. In C_4_ NAD-ME- and PCK-type species, rates of leaf *R*_dark_ are strongly correlated with organic acids, whereas in C_3_ and C_4_ NADP-ME-type species, *R*_dark_ is closely associated with substrates other than organic acids.

## Results

### Variation in leaf physiological traits

**Figure 1:**
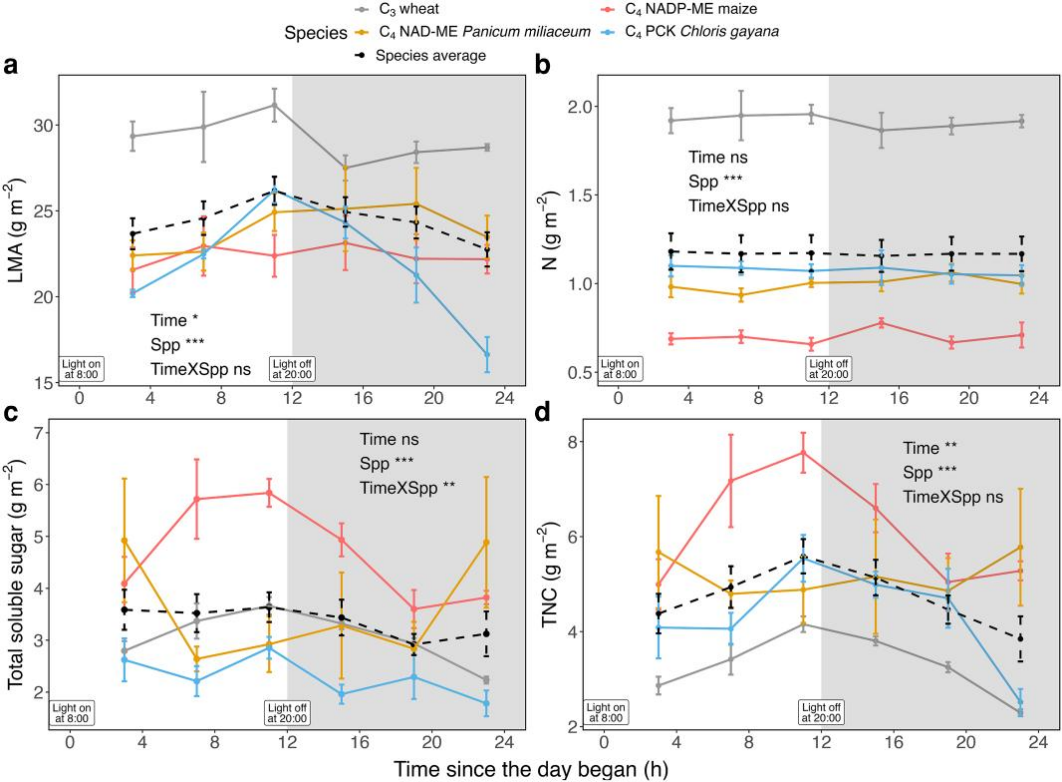
Physiological leaf traits of C_3_ and C_4_ species expressed per leaf area over a 24-h period. a) LMA; b) leaf N content; c) total soluble sugar; d) total nonstructural carbohydrates (TNC). Colored solid lines denote values of physiological leaf traits for each species, while black dashed lines indicate the averaged trait values across the 4 species. Unshaded and shaded regions denote day and night, respectively. Data are presented as mean ± SE at each measuring time point. Statistical results of a 2-way ANOVA examining time and/or species (Spp) effect are indicated on the figures (*P < 0.05; **P < 0.01; ***P < 0.001; ns, not significant) and presented in Table S1. Total biological sample sizes for a) to d) are 137, 142, 137, and 138, respectively.

Leaf mass per area (LMA) and N concentration differed among the 4 species (Fig. 1a and b; Table S1), with C_3_ wheat exhibiting significantly higher LMA values than the 3 C_4_ species at any given time (*P* < 0.001; Fig. 1a). LMA also varied significantly with time across all species (*P* < 0.05; Fig. 1a). Although there was no significant species × time interaction, the difference between LMA at sunset (around 11 h since the day began) and sunrise (around 23 h) was greatest in *Chloris gayana* (Fig. 1a). By contrast, leaf [N] expressed per unit leaf area was stable through time in all species (Fig. 1b). Wheat exhibited consistently higher leaf [N] than the other species (*P* < 0.001), followed by *C. gayana*, *Panicum miliaceum*, and maize, with leaf [N] of maize being significantly lower than all other species (*P* < 0.001).

**Figure 2:**
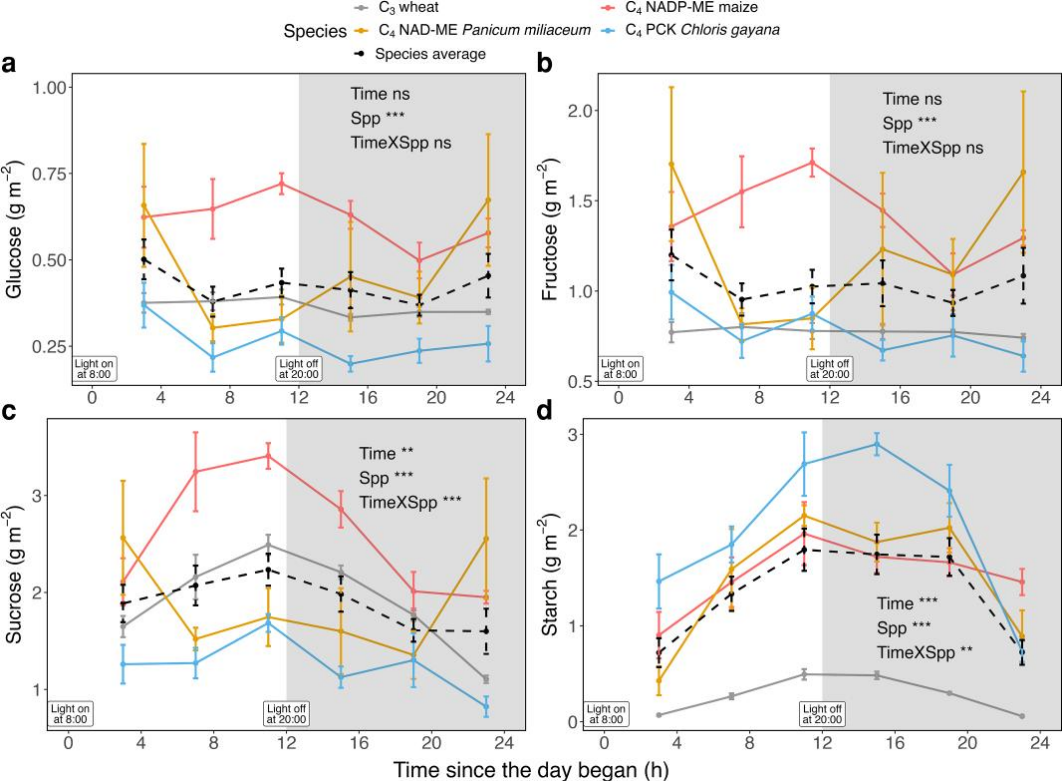
Soluble sugar and starch concentrations of C_3_ and C_4_ species expressed per leaf area over a 24-h period. a) Glucose; b) fructose; c) sucrose; d) starch. Colored solid lines denote values of soluble sugar and starch concentrations for each species, while black dashed lines indicate the averaged concentrations across the 4 species. Unshaded and shaded regions denote day and night, respectively. Data are presented as mean ± SE at each measuring time point. Statistical results of a 2-way ANOVA examining time and/or species (Spp) effect are indicated on the figures (*P < 0.05; **P < 0.01; ***P < 0.001; ns, not significant) and presented in Table S2. Total biological sample sizes for a) to d) are 137, 135, 136, and 132, respectively.

There was strong species-specific variation in leaf carbohydrates (Fig. 1c and d; Fig. 2; Table S2). Concentrations of total soluble sugars (ie sum of glucose, fructose, and sucrose) were similar among wheat, *P. miliaceum*, and *C. gayana*, but significantly higher in maize (*P* < 0.001; Fig. 1c). While there was no significant effect of time on total soluble sugars, a significant interaction effect of species and time was observed (Fig. 1c). This interaction effect was mostly attributed to the diurnal changes in sucrose in some species (Fig. 2c). Both wheat and maize showed a marked increase in sucrose content by the end of the day, followed by a sharp decline overnight (*P* < 0.05; Fig. 2c). The diel-driven changes in sucrose were not observed in glucose or fructose (Fig. 2a and b). The diel pattern of nonstructural carbohydrates (TNC; the sum of total soluble sugar and starch contents) largely resembled that of the total soluble sugar content, again driven mostly by sucrose response to time (Figs. 1c and d and 2c). The significant main effect of time on TNC but not total soluble sugars was due to the consistent increase in starch content during the day and depletion in starch overnight across all species (Fig. 2d). Wheat had the lowest TNC at all time points (Fig. 1d), mostly due to its lower starch content compared to the other species (*P* < 0.001; Fig. 2d). By contrast, all C_4_ species exhibited a significant increase in starch content through the day (*P* < 0.001), while only *P. miliaceum* and *C. gayana* had a significant reduction in starch at late night (*P* < 0.01 for all comparisons; Fig. 2d). By sunrise, all C_4_ species retained about 25% of their peak starch pool, whereas wheat had near completely consumed its starch pool (Fig. 2d).

### Rates of leaf *R*_dark_ vary over a day

**Figure 3:**
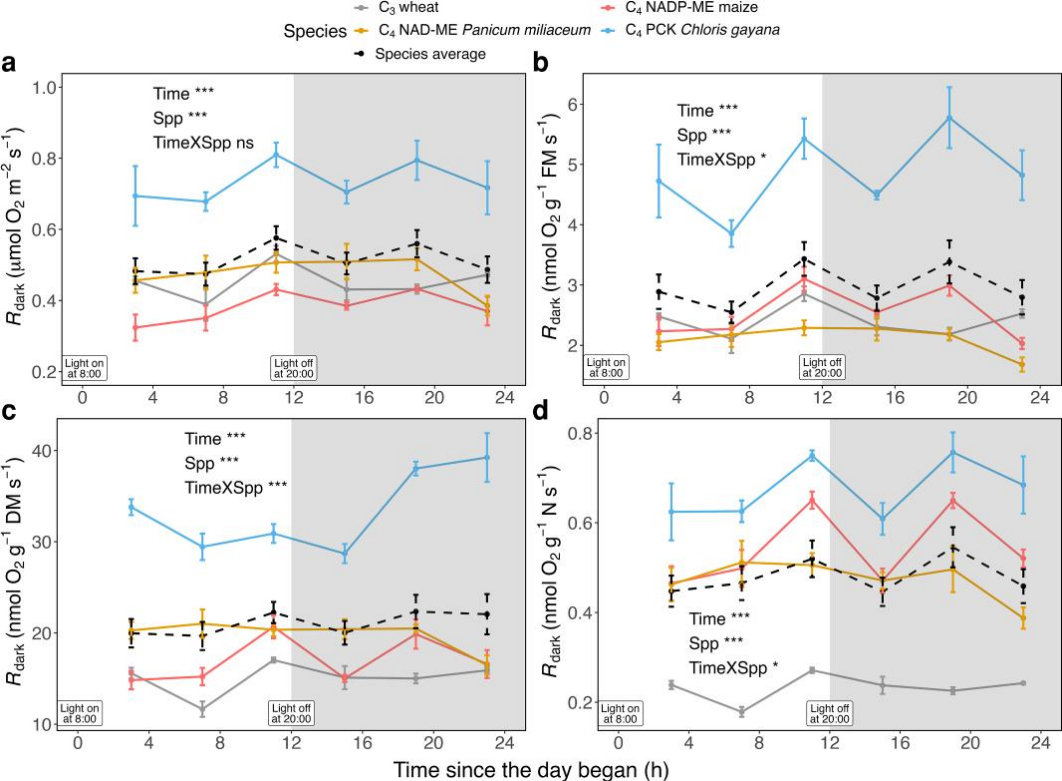
O_2_-based rate of leaf dark respiration (*R*_dark_) of C_3_ and C_4_ species expressed on 4 different units over a 24-h period. a) *R*_dark_ per leaf area; b) *R*_dark_ per leaf fresh mass (FM); c) *R*_dark_ per leaf dry mass (DM); d) *R*_dark_ per leaf N. Colored solid lines denote values of *R*_dark_ for each species, while black dashed lines indicate the averaged *R*_dark_ across the 4 species. Unshaded and shaded regions denote day and night, respectively. Data are presented as mean ± SE at each measuring time point. Statistical results of a 2-way ANOVA examining time and/or species (Spp) effect are indicated on the figures (*P < 0.05; **P < 0.01; ***P < 0.001; ns, not significant) and presented in Table S3. Total biological sample sizes for a) to d) are 137, 134, 137, and 138, respectively.

The O_2_-based rate of *R*_dark_ of *C. gayana* was significantly higher than that of the other species, regardless of whether it was relativized to leaf area, fresh/dry mass, or N content (Fig. 3; Table S3). While rates of O_2_-based *R*_dark_ in area and mass units were comparable among wheat, maize, and *P. miliaceum* (Fig. 3a to c), wheat exhibited significantly lower rates of *R*_dark_ than the other species when expressed per unit of [N] (Fig. 3d). This species-specific variation in *R*_dark_ was also confirmed using CO_2_-based measurements at 6 and 18 h since the day began (Figure S1). Irrespective of what basis *R*_dark_ was expressed on, a consistent main effect of time was found across the 4 species, indicating significant diel-driven fluctuation in *R*_dark_ (Fig. 3). However, the interaction between time and species was only observed for *R*_dark_ expressed on a mass basis (Fig. 3b to d). In *C. gayana*, *R*_dark_ per dry mass remained steady during the daytime but increased overnight (Fig. 3c); the night-dependent increase in *R*_dark_ per dry mass was likely a result of the associated decline in LMA at night in this species (Fig. 1a). Together, species-specific differences as well as the diel cycle both significantly affected *R*_dark_, with diel-driven variation in *R*_dark_ being more pronounced when changes in LMA over a day-night cycle (and associated changes in total nonstructural carbohydrates) were accounted for.

### Diurnal variations in metabolite profiles are linked to the accumulation of specific metabolites

**Figure 4:**
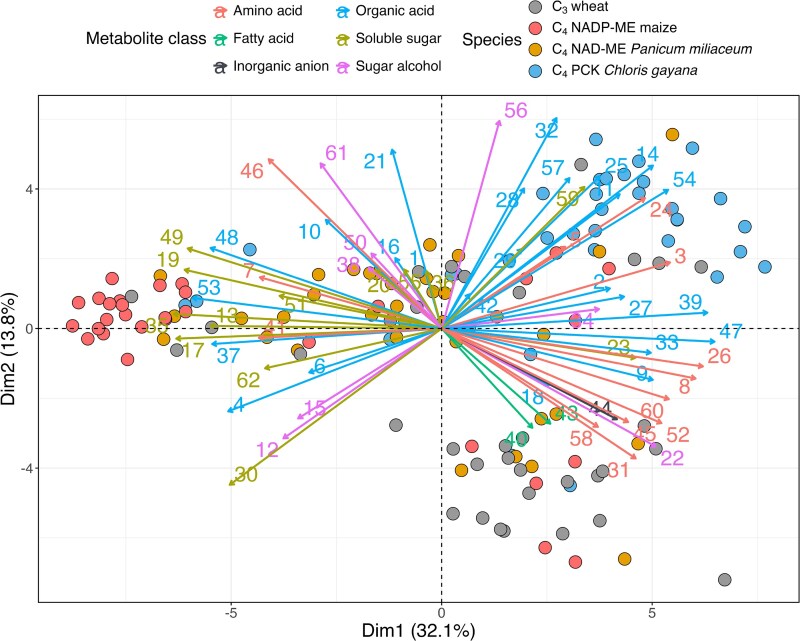
Biplots of PCA portraying relationships among metabolites in relation to C_3_ and C_4_ species over a day. Arrows, colored by metabolite classes, represent loading vectors indicating the contribution of individual metabolites to the sample separation. Points, colored by species, represent individual samples. See Table 1 for metabolite keys for loading arrows.

**Figure 5:**
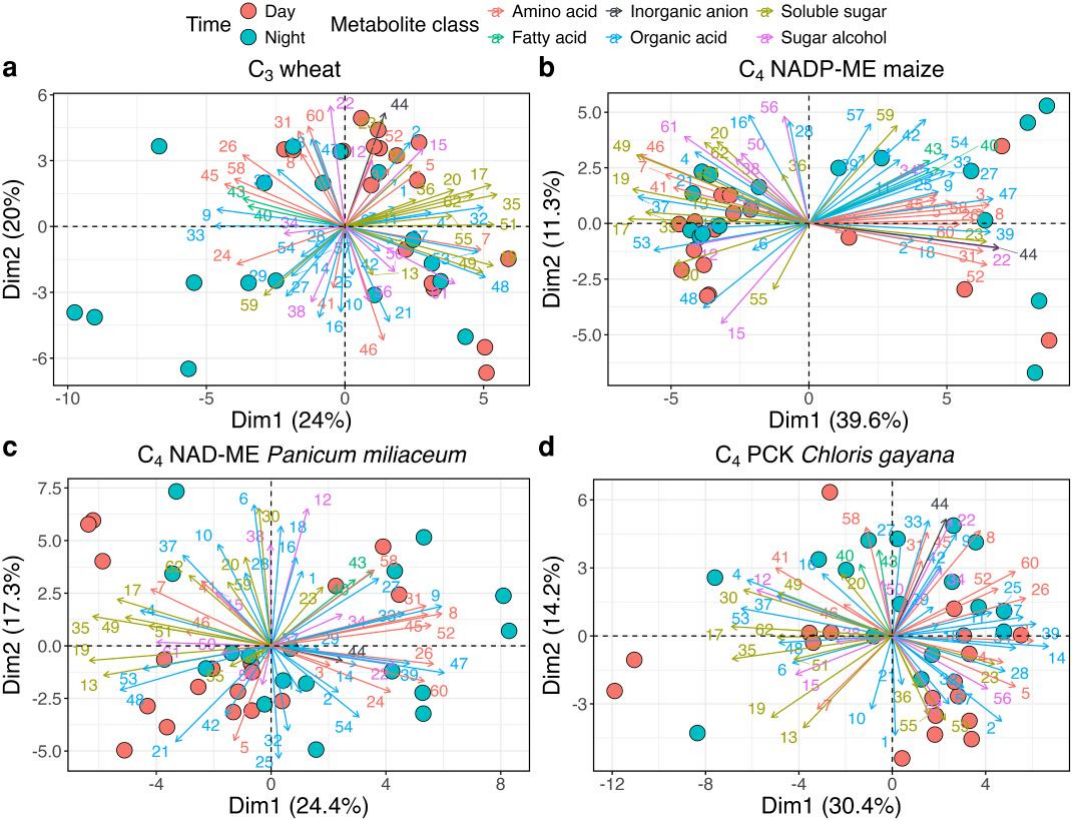
Biplots of PCA portraying relationships among metabolites in relation to the day-night cycle. a) C_3_ wheat, b) C_4_ NADP-ME maize, c) C_4_ NAD-ME *P. miliaceum*, and d) C_4_ PCK *C. gayana*. Arrows, colored by metabolite classes, represent loading vectors indicating the contribution of individual metabolites to the sample separation. Points, colored by sampling time point (day or night), represent individual samples. Day samples contain data collected at 3, 7, and 11 h, while night samples consist of data measured at 15, 19, and 23 h. See Table 1 for metabolite keys for the loading arrows.

**Figure 6:**
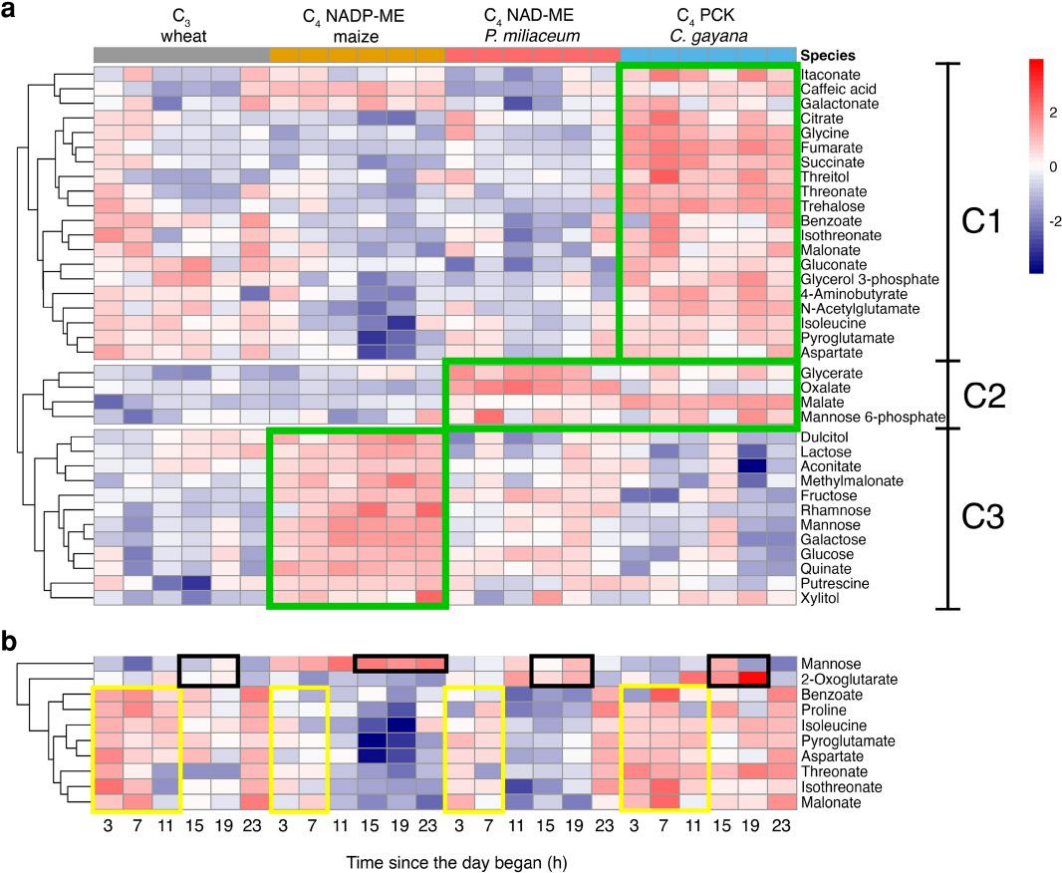
Hierarchical heatmaps contrasting relative levels of metabolites in dark-adapted C_3_ and C_4_ leaves. (a) Heatmap of metabolites showing significant differences among species (2-way ANOVA; *P* < 0.01). Three clusters are identified (labeled with C1, C2, and C3), and the most visible metabolite groups are framed with green boxes. (b) Heatmaps of metabolites associated with a significant time effect. The most visible metabolite changes during the day and at night are highlighted with yellow and black boxes, respectively. In both panels, metabolite levels were z-score transformed and scaled to make the range of values comparable between individual metabolites. The clustering was performed using the Complete method with Euclidean distance as a similarity measure. A full 2-way ANOVA table examining the effect of time and species can be found in Table 1.

We performed principal component analysis (PCA) to explore associations among metabolites in C_3_ and C_4_ species. There was a broad negative correlation between organic acids (blue arrows) and soluble sugars (yellow arrows), independent of species or day/night sampling time (Figs. 4 and 5; Table S4). C_4_ photosynthetic intermediates such as malate and aspartate, together with TCAP intermediates such as succinate and fumarate, showed strong positive correlation with one another and a strong negative correlation with soluble sugars such as mannose, lactose, glucose, and fructose (Fig. 4; Table S4). Strong species-specific differences in metabolite levels were observed. For example, metabolites that were abundant in maize grouped together at the opposite end (red dots on left-hand side of Fig. 4) of Dim 1 to *C. gayana* (blue dots on right-hand side), indicating a strong difference in organic acid to soluble sugar ratio between these 2 species (Fig. 4; also see Fig. 6a). *P. miliaceum* (orange dots) appeared to be intermediate between maize and *C. gayana*, while wheat (scattered gray dots) was not metabolically separated from the other 3 species.

When species were considered separately, metabolite differences between the day and night were visible with a PCA, particularly in wheat, and to a lesser extent in *P. miliaceum* and *C. gayana* (Fig. 5). Accumulation of soluble sugars during the day and organic acids at night were the main drivers of the dichotomy between day and night. Mannose, ribose, and glucose were the top 3 contributors to soluble sugar accumulation during the day in wheat (Table S5). By contrast, carbon intermediates involved in the TCAP, including fumarate, were driving the accumulation of organic acids at night in wheat and *P. miliaceum* (Fig. 5a and c). Maize had no clear separation of day or night metabolite profile (Fig. 5b).

### Leaf metabolome was influenced more by species than time

To further investigate the impact of time and species on individual metabolites, we conducted a 2-way ANOVA (with species and time as factors) to identify significant metabolites. Overall, the species effect was stronger than the time effect. Of the 62 metabolites reported, 35 were significantly influenced by species-specific variation, while 11 were affected by the diel cycle (Table 1). This variation among species was mainly attributed to higher levels of organic acids in *P. miliaceum* and *C. gayana* (Fig. 6a; Clusters 1 and 2), and higher levels of several sugars in maize (Fig. 6a; Cluster 3), compared to the other species. In general, wheat exhibited relatively lower contents of metabolites (Clusters 2 and 3) compared to the C_4_ species (Fig. 6a). However, it is worth noting that in wheat, maize, and *P. miliaceum*, several metabolites in Cluster 1 (eg aspartate and isoleucine) were more abundant at 3 h since the day began (indicated by yellow boxes in Fig. 6b) and gradually declined over time. Interestingly, the metabolites in Cluster 1 peaked again at 23 h since the day began in wheat and *P. miliaceum* (Fig. 6b), highlighting a potential increase in amino acid metabolism and sugar interconversions (Figure S2). This is further supported by elevated levels of mannose and 2-oxoglutarate at 15 and 19 h since the day began (indicated by black boxes in Fig. 6b). There was no time × species interaction on individual metabolites (Table 1). Overall, leaf metabolomes were more affected by species-specific differences, rather than by variation related to photosynthetic types (ie C_3_ vs. C_4_). Notably, many metabolites showing a species or time effect are involved in processes that release CO_2_ or consume O_2_ via NADH reoxidation (Table 1). These include 4-aminobutyrate (GABA shunt), gluconate (oxidative pentose phosphate pathway), putrescine and *N*-acetylglutamate (arginine/ornithine decarboxylation), benzoate (tyrosine degradation), and malonate (acetyl-CoA carboxylation) (Fig. 6b; Figure S2). Changes in these metabolites are likely to influence respiratory gas exchange.

**Table 1:** Two-way ANOVA of time and species effects on the metabolite level of C_3_ and C_4_ leaves.

				*P* value		
Key	Metabolite	Class	Pathway	Time	Species	Time × species
1	2-Hydroxyglutarate	Organic acid	Lysine degradation	0.105	0.013	0.025
2	2-Oxoglutarate	Organic acid	TCAP	0.006*	0.014	0.024
3	4-Aminobutyrate	Amino acid	GABA shunt	0.874	0.001*	0.256
4	Aconitate	Organic acid	TCAP	0.378	0.002*	0.573
5	Alanine	Amino acid	Amino acid metabolism	0.272	0.014	0.416
6	Ascorbate	Organic acid	Ascorbate metabolism	0.997	0.013	0.919
7	Asparagine	Amino acid	Aspartate metabolism	0.076	0.635	0.400
8	Aspartate	Amino acid	Aspartate metabolism	0.000*	0.002*	0.268
9	Benzoate	Organic acid	Tyrosine degradation	0.008*	0.009*	0.462
10	Caffeic acid	Organic acid	Phenylpropanoids	0.705	0.000*	0.496
11	Citrate	Organic acid	TCAP	0.045	0.003*	0.842
12	Dulcitol	Sugar alcohol	Galactose metabolism	0.328	0.000*	0.224
13	Fructose	Soluble sugar	Sugar interconversions	0.053	0.000*	0.159
14	Fumarate	Organic acid	TCAP and others	0.621	0.000*	0.992
15	Galactinol	Sugar alcohol	Galactose metabolism	0.391	0.279	0.391
16	Galactonate	Organic acid	Galactose metabolism	0.019	0.009*	0.368
17	Galactose	Soluble sugar	Galactose metabolism	0.012	0.000*	0.886
18	Gluconate	Organic acid	OPPP and aldarate metabolism	0.557	0.002*	0.687
19	Glucose	Soluble sugar	Sugar interconversions	0.017	0.000*	0.435
20	Glucose 1-phosphate	Soluble sugar	Hexosyl transferases	0.022	0.088	0.126
21	Glycerate	Organic acid	Photorespiration or cytoplasmic serine synthesis	0.158	0.000*	0.435
22	Glycerol	Sugar alcohol	Lipid metabolism	0.972	0.036*	0.147
23	Glycerol 3-phosphate	Soluble sugar	Lipid metabolism	0.060	0.001*	0.214
24	Glycine	Amino acid	Photorespiration or C_1_ metabolism	0.031	0.000*	0.244
25	Isocitrate	Organic acid	TCAP	0.505	0.060	0.956
26	Isoleucine	Amino acid	BCAA pathway	0.003*	0.000*	0.131
27	Isothreonate	Organic acid	Ascorbate metabolism	0.000*	0.006*	0.410
28	Itaconate	Organic acid	C_5_ branched acid pathway	0.170	0.002*	0.456
29	Lactate	Organic acid	Pyruvate metabolism	0.196	0.265	0.913
30	Lactose	Soluble sugar	Galactose metabolism	0.244	0.001*	0.647
31	Leucine	Amino acid	BCAA pathway	0.081	0.012	0.939
32	Malate	Organic acid	TCAP and others	0.649	0.000*	0.690
33	Malonate	Organic acid	Lipid metabolism	0.000*	0.001*	0.403
34	Mannitol	Sugar alcohol	Sugar interconversions	0.021	0.379	0.480
35	Mannose	Soluble sugar	Sugar interconversions	0.009*	0.000*	0.974
36	Mannose 6-phosphate	Soluble sugar	Sugar interconversion	0.553	0.099	0.218
37	Methylmalonate	Organic acid	BCAA pathway	0.946	0.001*	0.801
38	Myoinositol	Sugar alcohol	Galactose metabolism	0.083	0.397	0.445
39	*N*-Acetylglutamate	Organic acid	Arginine metabolism	0.809	0.000*	0.256
40	Octadecanoate	Fatty acid	Lipid metabolism	0.285	0.376	0.698
41	Ornithine	Amino acid	Arginine metabolism	0.617	0.027	0.973
42	Oxalate	Organic acid	Dicarboxylate metabolism	0.728	0.000*	0.874
43	Palmitate	Fatty acid	Lipid metabolism	0.109	0.321	0.892
44	Phosphate	Inorganic anion	n/a	0.789	0.055	0.098
45	Proline	Amino acid	Glutamate metabolism	0.002*	0.019	0.866
46	Putrescine	Amino acid	Arginine metabolism	0.016	0.010	0.537
47	Pyroglutamate	Organic acid	Glutamate metabolism	0.006*	0.000*	0.104
48	Quinate	Organic acid	Shikimate pathway	0.403	0.000*	0.993
49	Rhamnose	Soluble sugar	Sugar interconversions	0.542	0.001*	0.872
50	Ribonate	Sugar alcohol	OPPP and aldarate metabolism	0.414	0.600	0.765
51	Ribose	Soluble sugar	OPPP and aldarate metabolism	0.356	0.323	0.780
52	Serine	Amino acid	Photorespiration and C_1_ metabolism	0.599	0.013	0.595
53	Shikimate	Organic acid	Shikimate pathway	0.931	0.011	0.958
54	Succinate	Organic acid	TCAP	0.036	0.000*	0.672
55	Sucrose	Soluble sugar	Sugar interconversions	0.117	0.742	0.802
56	Threitol	Sugar alcohol	Sugar interconversions	0.188	0.002*	0.961
57	Threonate	Organic acid	Ascorbate metabolism	0.001*	0.000*	0.106
58	Threonine	Amino acid	Aspartate metabolism	0.058	0.343	0.376
59	Trehalose	Soluble sugar	Sugar interconversions	0.031	0.000*	0.088
60	Valine	Amino acid	BCAA pathway	0.018*	0.012	0.539
61	Xylitol	Sugar alcohol	OPPP and aldarate metabolism	0.824	0.004*	0.056
62	Xylose	Soluble sugar	OPPP and aldarate metabolism	0.041	0.063	0.147

Asterisk (*) indicates significance (*P* < 0.01). Time is considered as a continuous factor, while species is a discrete factor. Hierarchical heatmaps of significant metabolites can be found in Fig. 6, and a pathway map indicating the roles of metabolites can be found in Figure S2. The various sugars and polyols non-related to galactose are grouped into the broad pathway “sugar interconversions” for simplicity (except for pentoses and pentitols that are related to the OPPP). Although ascorbate comes from galactose, it is here differentiated in a separate group to make apparent the involvement of the ascorbate pathway. “Dulcitol” refers to galactitol (galactose-derived polyol). Total biological sample size = 135.

BCAA, branched chain amino acids; OPPP, oxidative pentose phosphate pathway; TCAP, mitochondrial tricarboxylic acid pathway.

### Correlations between metabolites and *R*_dark_

**Figure 7:**
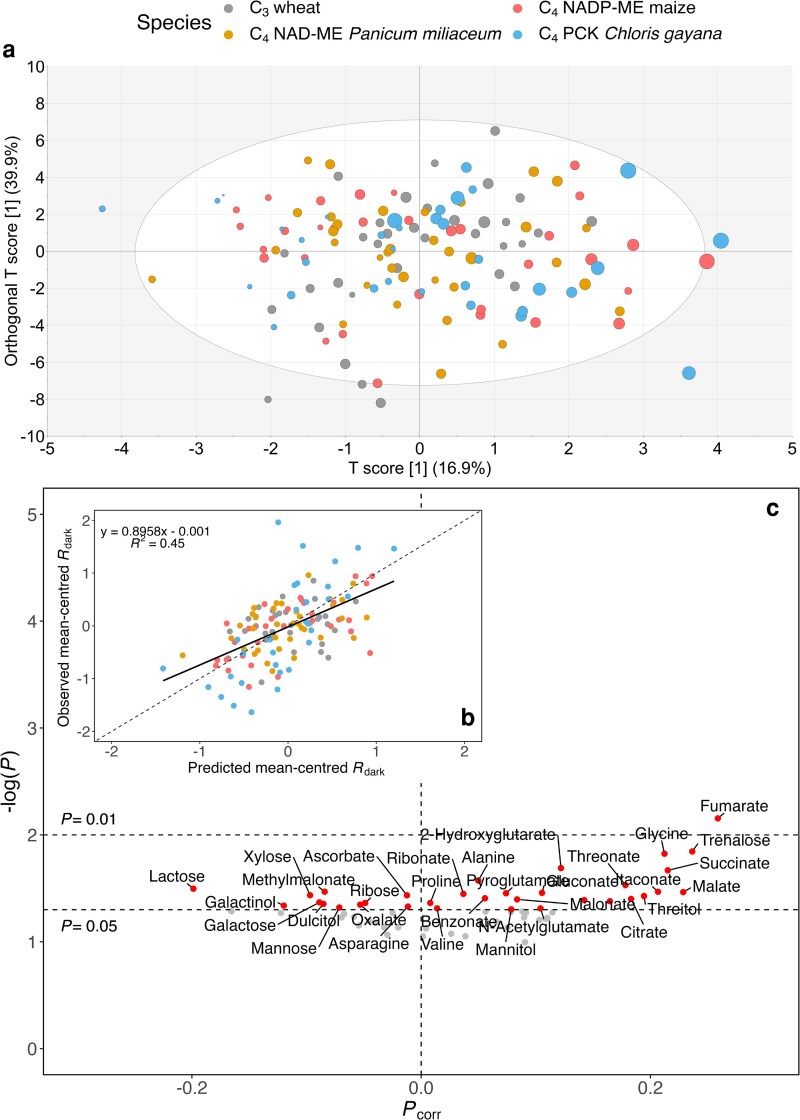
Multivariate OPLS analysis of metabolites with leaf dark respiration (*R*_dark_) expressed per unit of fresh mass as a response variable. a) Score plot of the OPLS colored by species, with the size of the discs reflecting rates of *R*_dark_ (ie faster the rate, bigger the disc size). b) Relationship between observed *R*_dark_ and OPLS-predicted *R*_dark_. The linear regression equation and *R*^2^ values are shown on the plot. The dashed line indicates a 1:1 ratio between observed and predicted *R*_dark_. c) Volcano plot illustrating the most correlated metabolites with *R*_dark_. The *y* axis, -log(*P*), indicates the -log of *P* values of a 1-way ANOVA testing relationship between individual metabolites and *R*_dark_, while the *x* axis *P*_corr_ denotes the loading of the OPLS. Only metabolites with *P* < 0.05 were labeled in c). Metabolites and *R*_dark_ data are mean-centered for each species to account for species-specific variation.

We next performed a quantitative orthogonal partial least squares (OPLS) analysis to examine what metabolites best correlated with variations in leaf *R*_dark_. Given that metabolome analysis was done on a fresh mass basis (ie using snap-frozen fresh leaves), we used leaf *R*_dark_ expressed per unit of fresh mass as the response variable *Y*. To prevent skewing of the model due to significantly higher *R*_dark_ rates and metabolite levels in *C. gayana* (Figs. 3 and 6a), we ran the model on mean-centered metabolite and *R*_dark_ data (ie z-scaling separately for each species). The OPLS model discriminated samples mostly along the *x* axis, with *C. gayana* and maize being the major contributor to sample discrimination along horizontal axis 1 (Fig. 7a). The model was statistically significant (*P*_CV-ANOVA_ = 8.5 × 10^−10^; *R*^2^ = 0.38, *Q*^2^ = 0.32), despite a few samples of *C. gayana* and maize that were outside the Hotelling's ellipse (Fig. 7a). This model fit was further reflected by the linear relationship between observed and model-predicted mean-centered *R*_dark_ (Fig. 7b). It is important to note that samples of *C. gayana* displayed a distinct difference in the slope between the observed and model-predicted *R*_dark_ (*y* = 1.514*x* + 0.004; Fig. 7b), likely due to its significantly higher *R*_dark_ rates in this species (Fig. 3). The loading of metabolites in OPLS (ie *P*_corr_ in Fig. 7c) suggests a negative correlation between organic acids (with *P*_corr_ > 0) and many sugars (with *P*_corr_ < 0). This result reinforces the metabolite correlations reported by PCA (Figs. 4 and 5). Importantly, the abundance of TCAP intermediates (eg fumarate, succinate, malate and citrate) was positively correlated with *R*_dark_, with fumarate being the best predictor of *R*_dark_. On the other hand, sugars (in particular lactose and galactinol) were negatively correlated with *R*_dark_ (Fig. 7c; Figure S3). There was little evidence that sample discrimination was driven by sampling time in the OPLS model (Figure S3). Taken together, regardless of the species and sampling time, the rate of *R*_dark_ appeared to be mostly driven by the balance between sugar consumption by catabolism via the TCAP and sugar interconversions via galactose metabolism.

## Discussion

### LMA dynamic in C_4_ PCK-type *C. gayana*

We found that LMA in *C. gayana* decreased markedly overnight, falling from 26.2 to 16.6 g m^−2^, a decline of 9.6 g m^−2^ (Fig. 1a). This result is surprising, as LMA is rarely reported to fluctuate to this extent over a 24-h diel cycle, even though it varies substantially across plant functional types, seasons, and biomes (Poorter et al. 2009). We argue that this diel variation in LMA could partially be attributed to a coupled decline in nonstructural carbohydrate reserves. In *C. gayana*, starch content declined from 2.9 to 0.7 g m^−2^ overnight, accounting for 2.2 g m^−2^ of the LMA decrease (Fig. 2d). Furthermore, total soluble sugars (the sum of glucose, fructose, and sucrose) fell from 2.3 to 1.8 g m^−2^, likely contributing to an additional 0.5 g m^−2^ decline in LMA (Fig. 1c). Our results also revealed significant diurnal variation in the concentrations of several organic acids (Fig. 6b), which may further contribute to diel changes in LMA. Future work is needed to elucidate the mechanisms underlying diel shifts in LMA in *C. gayana* and to determine whether similar diurnal patterns occur in other C_4_ PCK-type species.

### Soluble sugar dynamics in C_3_ and C_4_ plants

Our results show that in maize, there were significantly higher sucrose levels across nearly all time points (Fig. 2c). We suggest this may reflect a high initial partitioning of photosynthates to sucrose. In C_4_ leaves, sucrose is predominantly synthesized in mesophyll cells, while starch is formed in bundle sheath cells (Furbank et al. 1985; Lunn and Hatch 1995; Lunn and Furbank 1997, 1999; Furbank and Kelly 2021). It has been shown that the proportion of photosynthates initially partitioned to sucrose (compared to glucose, fructose or starch) is 3 to 4 times higher in NADP-ME-type leaves than in those of NAD-ME and PCK types (Lunn and Hatch 1995). Accordingly, sucrose levels in maize increased more rapidly than in *P. miliaceum* and *C. gayana* as the day progressed (Fig. 2c), even when *C. gayana* showed a 2-fold higher photosynthetic rate (Figure S1d). However, other studies have found similar (Lunn and Hatch 1995) or lower (Fan et al. 2024) daytime sucrose levels in NADP-ME-type species other than maize compared to NAD-ME and PCK types. These results highlight species-specific variation within a C_4_ biochemical type, and that measurements of sucrose levels (or more generally, carbohydrate levels) in whole leaves are a poor indicator of changes in carbohydrate fluxes (eg partitioning of photosynthate, conversion of sucrose to starch, sucrose export) (Lunn and Hatch 1995; Asao et al. 2025). The effect of high sucrose concentrations on sucrose transport in maize leaves is discussed in Note S1.

### Comparing key metabolite pools in C_3_ and C_4_ plants

Our study provides a time-resolved metabolic profile and *R*_dark_ values after 30 min of dark adaptation. As such, it offers insights into the metabolic properties of C_3_ and C_4_ species at each time point. For key C_4_ metabolites, relative malate levels (Cluster 2) remained significantly higher throughout the day than night in *P. miliaceum* and *C. gayana*, but not in wheat or maize (Fig. 6a). Similarly, relative aspartate levels (Cluster 1) were consistently increased in *C. gayana*, while in *P. miliaceum* and wheat aspartate showed transient increases at specific time points: 3, 7, and 23 h since the day began for *P. miliaceum* and 3, 7, and 15 h for wheat (Fig. 6a). The aspartate pattern is consistent with studies using light-harvested samples, which show that increased aspartate abundance is characteristic of C_4_ NAD-ME- and PCK-type species, but not of the NADP-ME type (Hatch 1987; Leegood and von Caemmerer 1988; Arrivault et al. 2017). However, our malate quantification results contrast with earlier reports, especially for wheat and maize. For example, an analysis of light-harvested samples in the genus *Flaveria* reported that the C_4_ NADP-ME-type *F. bidentis* and *F. trinervia* had at least 4-fold higher absolute malate content than C_3_  *Flaveria* species (Borghi et al. 2022). This discrepancy may be explained by the 30-min dark adaptation, which could have altered daytime metabolism and stimulated cytosolic malate-consuming processes (eg malate decarboxylation via TCAP or enhanced vacuolar malate storage).

Beyond C_4_ metabolism, our comparison of dark-adapted levels of key primary metabolites (ie glycine, citrate, aconitate, and 4-aminobutyrate; Fig. 6a) between wheat and maize is broadly consistent with metabolomic studies comparing C_3_ and C_4_ NADP-ME-type species in genus *Flaveria*, using samples collected in the light (Borghi et al. 2022; Tang et al. 2024). Notably, these metabolite levels were significantly higher in *C. gayana*, suggesting increased activity in the cytosolic serine pathway (involving glycine), the TCAP (citrate and aconitate), and the GABA shunt (4-aminobutyrate) (Figure S2). These metabolic signatures may also underpin high rates of *R*_dark_ observed in this species (see below).

### Differences in leaf *R*_dark_ among species

Past studies have reported diel-driven fluctuations in rates of *R*_dark_ in a range of C_3_ and C_4_ species, including using O_2_-based measurements of *R*_dark_ in rice (Rashid et al. 2020) and CO_2_-based *R*_dark_ measurements in sugarcane (De Souza et al. 2018), cotton (Gessler et al. 2017), and bean (Gessler et al. 2017). In our study, while rates of O_2_-based *R*_dark_ did vary with time, there was no striking diel pattern across the 4 species, when the rates were expressed per unit leaf area, fresh mass or leaf nitrogen (Fig. 3). Only when expressed on a dry mass basis did one species (C_4_ PCK-type *C. gayana*) exhibit marked diel shifts, with *R*_dark_ rising sharply toward the end of the night (Fig. 3c). This shift corresponded to a marked decline in dry mass per unit leaf area (ie LMA) and starch content of *C. gayana* (Figs. 1a and 2d), suggesting that changes in LMA and TNC may partially contribute the change in *R*_dark_ rather than alterations in respiratory metabolism (ie O_2_ consumption) per se.

Comparison of O_2_-based *R*_dark_ across the 4 species revealed marked differences in overall respiratory rates, with *C. gayana* having the highest *R*_dark_ regardless of units (Fig. 3). Since our study used mature leaves, we propose that these differences in *R*_dark_ were associated with differences in maintenance respiration, possibly through providing ATP and carbon skeletons for protein turnover and sucrose export. It has been reported that a C_4_ PCK-type species, *Urochloa panicoides*, exhibited the highest rate of *R*_dark_ among species of the 3 C_4_ types in a substrate-saturated and non-adenylate-limited condition in bundle sheath strands (Agostino et al. 1996). Such a high rate may also result from a high enzymatic capacity to process respiratory substrates, yet we observed similar respiratory capacity in C_4_ NAD-ME-type *P. miliaceum* and PCK-type *U. panicoides* (Fan et al. 2022b). Considering the interspecific variation among PCK-type species (eg *U. panicoides* vs. *C. gayana*), the high rate of *R*_dark_ seen in *C. gayana* (Fig. 3) may be reflective of high respiratory product demand to maintain cellular processes, knowing that the plants were likely not under any substrate limitation (Fig. 4). This view is to some extent supported by the values of *R*_dark_ per leaf [N] in *C. gayana* (Fig. 3d), which indicate that the ATP cost for maintaining a unit of leaf protein was the highest in this species. Our previous and current studies highlight that diel-driven variation is often more pronounced in CO_2_-based (Figure S1) than O_2_-based *R*_dark_ (Fig. 3) in a species-specific manner (Fan et al. 2024).

### Is dark-adapted daytime *R*_dark_ similar to nighttime *R*_dark_?

Our study shows that dark-adapted starch, soluble carbohydrates, and other metabolites related to respiratory metabolism vary through a diel cycle in selected C_3_ and C_4_ species. Importantly, diel patterns in these traits were highly dynamic and depended on species and were associated with concurrent changes in *R*_dark_ itself (Figs. 1 to 5). The regulation of starch degradation is further discussed in Note S2. One of the most notable metabolic responses was that soluble sugars accumulated during the day and organic acids at night (Figs. 5 and 6).

Our metabolomics analysis suggests there was a negative correlation between soluble sugars and organic acids, independent of sampling period and species (Figs. 4 and 5). This is different from the negative correlation between amino acids and sugars that Rashid et al. (2020) observed in day- and night-sampled rice leaves. The difference may result from active protein synthesis and N assimilation in the light. We note that in Rashid et al. (2020), samples were illuminated during the day sampling period and were measured for metabolite profile without the 30-min dark adaptation used in our study. Past studies have reported that dark adaptation has a profound effect on a large number of metabolites (Urbanczyk-Wochniak et al. 2005; Gessler et al. 2009). Together, a 30-min dark adaptation is likely to fundamentally change daytime metabolism, supporting findings in Florez-Sarasa et al. (2012). In addition, our results point to 30-min dark adaptation being insufficient to eliminate the differences in leaf metabolism between dark-adapted day- and night-harvested samples (Figs. 5 and 6b and  Table 1). This is likely due to the slow progression of post-illumination processes, such as light-enhanced dark respiration and adjustment of metabolic pools to a level equivalent to dark metabolism, which together may take longer than 30 min (Barbour et al. 2007; Gessler et al. 2009). This should affect *R*_dark_ measurements in routine gas exchange experiments. Specifically, the common assumption that exposure to darkness for 30 min allows leaves to shift from day to night metabolism (Azcón-Bieto et al. 1983; Stitt et al. 1985; Atkin et al. 2000; Padmasree et al. 2002) appears to be invalid, at least for the species studied here. In addition, our study shows that *R*_dark_ fluctuates with time of day, highlighting the importance of selecting appropriate sampling times and ensuring sufficient temporal resolution to obtain representative estimates of diel changes in leaf traits.

### How many sampling points are sufficient to capture diel variation in *R*_dark_?

Our results on diel variation in O_2_-based *R*_dark_ challenge the common practice of using a single daytime, dark-adapted measurement to represent *R*_dark_ across the entire diel cycle. O_2_-based *R*_dark_ has previously been reported to fluctuate over a diel cycle (Rashid et al. 2020), and CO_2_-based *R*_dark_ can decline overnight due to a shift in the type of respiratory substrates used (Bruhn et al. 2022, 2025; Bruhn 2024; Jones et al. 2024). These findings highlight the need to closely monitor continuous changes in *R*_dark_ across a diel cycle. While continuous daytime sampling is feasible, albeit labor-intensive, nighttime sampling poses significant safety challenges in both laboratory and field settings. It has been suggested that the overnight decline in CO_2_-based *R*_dark_ can be estimated by applying a linear regression-based scaling relative to the measurement taken around sunset (Bruhn et al. 2024b). This alleviates the need to sample at night if the research objective is to examine diel variation in CO_2_-based *R*_dark_. As for O_2_-based *R*_dark_, we suggest that sampling at a single time point (ie around sunset) would be sufficient to give a reasonable estimate of *R*_dark_ over a diel cycle (Fig. 3), provided that LMA and TNC remain stable. When LMA shows pronounced diel fluctuations (eg *C. gayana* in Fig. 1a), sampling at 2 time points (ie sunset and sunrise) would be needed to account for variation in O_2_-based *R*_dark_ expressed on a dry-mass basis (Fig. 3c). In summary, a single *R*_dark_ measurement around sunset is recommended, so long as LMA and TNC do not vary substantially over a diel cycle.

### *R*_dark_ reflects species-specific metabolic pathways beyond conventional “TCAP respiration”

Our metabolomics analysis showed that many organic and amino acids (fumarate, succinate, malate, itaconate, isoleucine, 4-aminobutyrate, and *N*-acetylglutamate) were higher in *P. miliaceum* and/or *C. gayana* compared to maize and wheat (Cluster 1 and 2 in Fig. 6a). From a perspective of C_4_ biochemistry, this result likely reflects malate (or more generally, C_4_ acids) recycling in *P. miliaceum* and *C. gayana* mitochondria to fuel *R*_dark_. C_4_ NAD-ME- and PCK-type species use malate and/or aspartate as C_4_ acids in their photosynthetic CO_2_-concentrating mechanisms, with decarboxylation of these acids occurring in the mitochondria or requiring mitochondrial ATP (von Caemmerer and Furbank 2016). As such, mitochondria in C_4_ NAD-ME and PCK species are exposed to daytime photosynthetic fluxes that are ∼10 times higher than those at night/in the dark, requiring elevated enzymatic capacity to decarboxylate the resulting C_4_ substrate flux (Hatch and Carnal 1992; Fan et al. 2022a). Studies have shown that in many C_4_ NAD-ME and PCK species of the PACMAD clade (including C_4_ species used in this study), the capacity for mitochondrial decarboxylation of C_4_ acids is retained in the dark (Fan et al. 2022b), and *R*_dark_ can be fueled by C_4_ acids (Agostino et al. 1996; Fan et al. 2024). Future work is needed to validate this *R*_dark_ mechanism across a wider range of species representative of NAD-ME and PCK types in C_4_ lineages beyond the PACMAD clade.

The use of malate also generates various TCAP intermediates (eg citrate, fumarate, succinate and 2-oxoglutarate) and pyruvate (via the malic enzyme), which can further fuel the C_5_ branched acid pathway (itaconate production), GABA shunt, and branched chain amino acids (BCAA) synthesis (Fig. 6a; Figure S2). It is worth noting that the GABA shunt functions as a partial bypass of the TCAP, producing CO_2_ and potentially playing a role in coordinating C and N metabolism when protein turnover demands remain unchanged (Bouché and Fromm 2004). Enhanced production of leucine and isoleucine involves the utilization of pyruvate (or acetyl-CoA), thereby providing reducing equivalents to the mitochondrial electron transport chain (mETC) (Taylor et al. 2004). Other significant metabolites (such as oxalate and benzoate) are catabolic products, the synthesis of which generates CO_2_ and reducing equivalents (NAD(P)H), feeding the mETC. In NADP-ME-type maize, metabolites associated with CO_2_ or NAD(P)H generation were found to be significant, including aconitate (a TCAP intermediate), putrescine, and methyl malonate. This finding is supported by significantly higher levels of citrate (a precursor of aconitate) and 2-oxoglutarate (a precursor of putrescine) in 2 C_4_ NADP-ME-type *Flaveria* species compared to their C_3_ counterparts (Borghi et al. 2022). Interestingly, in maize, there were also significantly higher levels of soluble sugars (eg galactose, dulcitol, rhamnose, mannose, and lactose) (Cluster 3 in Fig. 6a). The prevalence of galactose metabolism (generating lactose and dulcitol) and fructose interconversion (generating mannose and rhamnose) in maize may play a role in nighttime sugar recycling (Yesbergenova-Cuny et al. 2016), alleviation of redox stress and regeneration of antioxidants (Smirnoff 1996; Smirnoff and Wheeler 2000; Akram et al. 2017; Araniti et al. 2018), and defense mechanisms (Waterman 2007; Zaynab et al. 2018). Collectively, these findings suggest that the observed metabolic differences among C_3_ and C_4_ species reflect contrasting, species-specific strategies to balance metabolic imperatives (maintenance respiration) and support critical pathways (eg export to the phloem or stress tolerance), which, in turn, affect *R*_dark_.

### What are the substrates that best correlate with variations in *R*_dark_?

Multivariate OPLS analysis suggested that *R*_dark_ was positively related to the abundance of key metabolites, in particular TCAP intermediates (malate, fumarate, and succinate), and negatively correlated to sugars and derivatives of galactose metabolism (Fig. 7c). It is not surprising that TCAP intermediates were positively correlated with *R*_dark_ since oxidation of malate, fumarate, and succinate produces reducing equivalents, which in turn leads to O_2_ uptake via the mETC (Rasmusson et al. 2014). Our results align closely with a study in Arabidopsis, highlighting that changes in organic acids have a greater impact on leaf *R*_dark_ than soluble sugars (Florez-Sarasa et al. 2012). The present results support our previous suggestion that changes in malate and succinate availability were strongly correlated with the midday-to-midnight variation in *R*_dark_ (Fan et al. 2024). In addition, it has been recently shown via OPLS multivariate analysis that (*i*) both rates of *R*_dark_ and the natural ^13^C abundance in leaf-respired CO_2_ were mostly explained by the kinetics of organic acid (eg malate and citrate) turnover (Xia et al. 2024) and (*ii*) organic acids such as aconitate and citrate are the best predictors of the increase in *R*_dark_ rate in K-deficient plants, along with markers of aldarate metabolism (sugar oxidation to glucarate and 2-oxogluconate) (Cui et al. 2019). Interestingly, the best predictor of *R*_dark_ found here was fumarate (Fig. 7c). This probably reflects the fact that multiple metabolic pathways involve fumarate as an intermediate. Fumarate lies at the crossroad of different processes related to respiration and plays an important role in multiple pathways (Figs. 6 and 7; Figure S2): (*i*) TCAP (via succinate dehydrogenase, complex II), (*ii*) arginine and putrescine metabolism (via argininosuccinate lyase), and (*iii*) tyrosine degradation (which leads to benzoate, acetoacetate and fumarate as end products). Accordingly, isotope labeling has shown that there is an important change in the turnover of the joint fumarate-malate pool between daytime and nighttime (via the regulation of the TCAP, malic enzyme and PEP carboxylase), and this change influences the transition from day to night respiration (Abadie et al. 2024).

The negative correlation between galactose metabolism and *R*_dark_ (Fig. 7c) may appear surprising since our previous work suggested a strong positive correlation (Fan et al. 2024). However, in our previous work, there was limited time resolution (ie midday and midnight only). In our current study, with the better time resolution used, lactose showed species-specific diel fluctuations (Fig. 6a, Cluster 3). Additionally, 4 metabolites of galactose metabolism (lactose, galactose, galactinol, and dulcitol) were negatively correlated with *R*_dark_, along with sugars (xylose and mannose) in sugar alcohol metabolism (Figure S2). This observation suggests that in the examined species, higher activity of galactose metabolism was effectively associated with lower leaf *R*_dark_. While the origin of this phenomenon requires further work, one might hypothesize that it may be associated with the interplay between sugar interconversions, ascorbate synthesis from galactose, export to phloem, and sugar consumption by glycolysis. In wheat, isotopic labeling with ^14^C has shown that galactose is mostly interconverted to glucose and sucrose (Hassid et al. 1956). Possible interactions between leaf *R*_dark_ and ascorbate synthesis from galactose have been reviewed recently (Smirnoff and Wheeler 2024). Interestingly, in Arabidopsis, mutants lacking NAD-dependent malic enzyme showed no developmental phenotype but exhibited altered metabolism, most notably, increased galactose levels during the day, and decreased fumarate and increased glutamine levels at night (Tronconi et al. 2008). Collectively, several lines of evidence suggest that galactose metabolism may be negatively associated with *R*_dark_ and that (ga)lactose and fumarate are opposing indicators of *R*_dark_.

### Perspectives

We show that both leaf metabolites and *R*_dark_ varied in a species-specific manner, irrespective of the photosynthetic type (C_3_ or C_4_), and changed throughout a day. There were consistent diel differences, suggesting that daytime *R*_dark_ measured after 30-min adaptation was not a surrogate for nighttime *R*_dark_. O_2_-based *R*_dark_ likely resulted from multiple metabolic pathways, and *R*_dark_ could be estimated statistically with various metabolites, with fumarate being the best biomarker of *R*_dark_. Similarly, it has been recently shown that day respiration (non-photorespiratory CO_2_ evolution in the light) is the result of multiple metabolic pathways (Tcherkez et al. 2024), with some of them overlapping with pathways suggested here (such as BCAA metabolism). Further work (eg using ^13^C labeling) is needed to assess the quantitative impact of such pathways on overall respiratory O_2_ consumption or CO_2_ production. Additionally, since amino acid synthesis and degradation are involved, it is likely that the difference between daytime and nighttime (Figs. 5 and 6) and the quantitative relationship between metabolites with *R*_dark_ (Fig. 7) change under contrasting N conditions. It is also worth noting that the C_3_ and C_4_ species examined in this study were all grasses belonging to the PACMAD clade. It would be interesting to investigate whether similar patterns emerge in other taxonomic groups, such as *Flaveria* and *Tribulus*, and whether such patterns systematically occur in multiple C_4_ lineages.

## Materials and methods

### Plant material and sampling

C_3_  *T. aestivum* (wheat, cultivar Seri/Rayon), C_4_ NADP-ME *Zea mays* (maize, cultivar Delphine), C_4_ NAD-ME *P. miliaceum*, and C_4_ PCK *C. gayana* were grown from seeds in 6-L pots in organic potting soil supplemented with slow-release fertilizer (Scotts Osmocote Inc.). Plants were grown in a completely randomized order in controlled Climatron growth cabinets (Thermoline Inc.) and maintained at 30/25 °C day/night, 12/12 h light/dark (day started at 08:00) with 500 to 600 µmol m^−2^ s^−1^ photosynthetically active radiation (PAR) at plant height during the day and ambient CO_2_ concentration. The most recent fully expanded leaves of 5-week-old plants were used in all measurements.

Leaves were harvested from 6 individual plants per species at 3 (11:00), 7 (15:00), 11 (19:00), 15 (23:00), 19 (03:00), and 23 (07:00) hours since the day began and dark-adapted for 30 min. After a 30-min dark adaptation, the middle portion of the leaf was cut into 5 sections using a custom-made 2 × 3 cm metal cutting frame. The sections were labeled from section 1 (nearest the leaf tip) to section 5 (nearest the leaf base), with each section approximately 2 cm in length. Sections 1, 2, 4, and 5 were snap-frozen in liquid nitrogen within 10 s after the initial cut and stored at −80 °C. Section 3 (ie middle section of a leaf) was subsequently used for O_2_-based *R*_dark_ measurements and for determining LMA and total leaf [N] (see below). Frozen leaf sections 1 and 5 were later used in starch and soluble sugar analysis, while sections 2 and 4 were used in metabolite quantification. A total of 144 plants (4 species × 6 replicates × 6 time points) were sampled in this study.

### Measurements of photosynthesis and dark respiration rates

The third section of each leaf was used to measure O_2_-based *R*_dark_ using a fluorophore oxygen sensor (Astec Global, Maarssen, The Netherlands), as documented in O’Leary et al. (2017) and Scafaro et al. (2017). Leaf sections were first measured for leaf area and then placed into 2 mL air-tight tubes to monitor changes in O_2_ concentration. The assay was conducted at 30 °C in the dark for 3 h, and *R*_dark_ was calculated from the rate of O_2_ consumption recorded between 30 min and 3 h after the start of the measurement. After the assay, leaves were then dried for at least 2 d at 60 °C to determine dry mass and total leaf [N].

We also quantified CO_2_-based *R*_dark_ and photosynthesis at high light (1,500 µmol quanta m^−2^ s^−1^ light; termed as *A*_1500_) rates using a LI-COR 6400-XT infrared gas analyzer (Li-Cor BioSciences) in separate sets of plants at midday and midnight. At midday (6 h after lights on; 14:00), leaves from a new set of 4 to 6 individual plants per species were measured for *A*_1500_ at 500 µmol s^−1^ flow rate, with the LI-COR chamber temperature and sample CO_2_ concentration being set to 30 °C and 400 ppm, respectively. Thereafter, leaves were removed from the LI-COR chamber and wrapped in aluminum foil for 30 min of dark adaptation. The dark-adapted leaves were subsequently placed back into the LI-COR chamber, and CO_2_-based *R*_dark_ was measured (with lights off). At midnight (6 h after lights off; 02:00), leaves from another set of 4 to 6 individual plants per species were determined for CO_2_-based *R*_dark_ as described above. Leaf sections clamped by the LI-COR chamber were cut and oven-dried for at least 2 d at 60 °C, followed by determining dry mass.

### Leaf nitrogen, starch, and soluble sugar analysis

To analyze total leaf nitrogen concentration, dried leaves from gas exchange measurements were ground and placed into tin capsules for combustion analysis using a system combining a Heraeus Elementary CHN-O Rapid elemental analyzer (Döbeln Elektrowärme GmbH) for Dumas combustion of the samples, a Finnigan MAT Trapping box HT (Thermo Fisher Scientific Inc.) for automatic cryo-purification of the combustion products.

To analyze the leaf starch and soluble sugar contents, freeze-dried leaf sections were ground into fine powder, and 5 to 10 mg of the powder was placed in a 2-mL microfuge tube with 0.5 mL of 80% (v/v) ethanol to extract soluble sugar and starch. The tissue was vigorously vortexed for 20 s at 500 rpm and incubated on a Thermomixer orbital shaker at 80 °C for 20 min (Eppendorf South Pacific Pty Ltd.). The tissue was then centrifuged for 5 min at 16,260 *g*, and the supernatant was collected. The above extraction steps were further repeated twice on the remaining pellet, and the supernatant was pooled. Soluble sugar levels in the pooled supernatant and starch concentrations in the remaining pellet were determined using a fructose assay kit (catalog # FA20-1KT; Sigma-Aldrich Inc.) and a total starch assay kit (catalog # K-TSTA-100A; Megazyme Inc.), respectively, following the manufacturer's instructions and Rashid et al. (2020). Measurements were collected using a microplate reader (Infinite M1000Pro; Tecan Group Ltd.) at a wavelength of 340 nm for sugars or 515 nm for starch.

### Gas chromatography-mass spectrometry (GC-MS) metabolite analysis

GC-MS was used to quantify metabolite profiles of the sampled leaves over a day. Metabolite extraction was performed according to the procedure described in Che-Othman et al. (2020) with some modifications. Frozen leaf tissue was ground to powder, and approximately 25 mg of leaf powder was transferred into 2 mL microfuge tubes. After that, 0.5 mL of cold extraction buffer (1:2.5:1 [v/v/v] chloroform, methanol and water, 0.1% [v/v] L-valine-^13^C_6_ and D-sorbitol-^13^C_6_ internal standards) was added. The mixture was then incubated, vortexed, and centrifuged at 14,500 *g* at 4 °C for a total of 3 times, with supernatant pooled to a total of 1.5 mL. Then, 0.4 mL of HPLC-grade water was added to phase-separate, and the upper phase was collected and dried down in a SpeedVac vacuum concentrator (Thermo Fisher Scientific Inc.) at 30 °C overnight before metabolite profiling using GC-MS. Dried extracts were methoximated by adding 20 µL of a 20 mg mL^−1^ solution of methoxyamine hydrochloride in anhydrous pyridine (Sigma-Aldrich Inc.) and incubating at 30 °C for 90 min. For trimethylsilylation, 30 µL of N-methyl-N-(trimethylsilyl)trifluoroacetamide was added to each tube, followed by incubation at 37 °C for 30 min. Subsequently, 10 µL of an n-alkane retention index calibration mixture, containing 0.29% (v/v) n-dodecane, 0.29% (v/v) n-pentadecane, and 0.29% (w/v) each of n-nonadecane, n-docosane, n-octacosane, n-dotriacontane, and n-hexatriacontane in anhydrous pyridine, was added. The mixtures were vortexed, transferred to amber GC-MS vials with low-volume inserts and screw-top seals (Agilent Technologies), and left to rest for 4 h before GC-MS analysis. The derivatization was performed using the MPS2 XL-Twister autosampler (Gerstel GmbH & Co. KG, Mülheim an der Ruhr, Germany). Here, 1 µL of derivatized sample was immediately vaporized in the inlet at 250 °C and injected into the chromatography column in a split-less mode. Helium was used as the carrier gas. Compounds were eluted using the following temperature gradient: hold for 1 min at 70 °C then ramp at 7 °C min^−1^ to 325 °C and finally hold for 3.5 min. The ion transfer line was heated to 280 °C and the ion source and quadrupole were heated to 150 and 230 °C, respectively. The resulting peaks were analyzed using MS-DIAL software v.4.48 (Tsugawa et al. 2015). The height of the quantifier ion for each peak was compared between samples after normalization. Metabolites were normalized against the weighted and averaged signals of 2 internal standards and fresh leaf mass, followed by weighting against the average measured signal across all samples for each compound (ie z-score normalized) before statistical analyses were performed. All metabolite samples were randomly loaded onto GC/MS and independent of the time collected. Fresh mass was chosen as the unit for normalizing metabolite signals because metabolites were extracted from frozen fresh leaf tissue. We recognize that the results could differ if normalization were performed using a different unit, highlighting a persistent challenge in comparing species, mutants, or cultivars in metabolomics studies (van den Berg et al. 2006).

### Data transformation and statistical analysis

Gas exchange and leaf trait data were screened for outliers using the boxplot (interquartile range) method. Values lying below Q1 − 1.5 × IQR or above Q3 + 1.5 × IQR, where Q1 and Q3 represent the first and third quartiles and IQR is the interquartile range, were identified as outliers and removed prior to analysis. No additional scaling or transformation was performed on gas exchange and leaf trait data. By contrast, metabolite data were z-score transformed by normalizing against leaf fresh mass and internal metabolite standards (^13^C-valine and ^13^C-sorbitol).

All data were then analyzed using 2-way analyses of variance (ANOVA), PCA, hierarchical cluster analysis, multiple comparison tests, and OPLS multivariate analysis using R v.4.4.1 (R Core Team 2024) and Sartorius SIMCA 18 (Umetrics, Umeå, Sweden) (Cui et al. 2019). Each ANOVA model was fit using the *aov* function in R, which fits linear models by least squares. The models were specified with species, time, and their interaction term as independent variables, while the physiological and gas exchange traits measured were dependent variables (ie *y* axis parameters in Figs. 1 to 3 and individual metabolite contents in Table 1). The test statistics were derived from the *F*-ratio, calculated as the mean square of the dependent variables divided by the mean square of the residuals. *P* values were obtained from the corresponding *F*-distribution. For each effect, the degrees of freedom (df) corresponded to the number of levels of the independent variables minus one.

Prior to PCA and OPLS multivariate analyses, the z-score transformed metabolite data were further log-transformed to reduce heteroscedasticity and right-skewness typical of concentration datasets (Dataset S1). PCA and hierarchical cluster analysis were fitted using R packages of *factoextra*, *FactoMineR*, *NbClust*, and *cluster*. In PCA, the individuals were the biological samples from 4 species collected at 6 time points, and the variables were the metabolite contents. The correlation between leaf *R*_dark_ and metabolite profiles was examined using OPLS multivariate analysis as described by Cui et al. (2019). The OPLS analysis considered metabolite concentrations and *R*_dark_ as predicting (*X*) and quantitative response (*Y*) variables, respectively. The correlation between each detected metabolite and *R*_dark_ was shown in a volcano plot combining the output of the OPLS (ie loading on the *x* axis) and -log(*P*) obtained via an ANOVA, with the best correlating metabolites being labeled. All comparisons were considered significant at *P* < 0.05, unless stated otherwise in figures.

## Supplementary Material

kiag200_Supplementary_Data

## Data Availability

The data that support the findings of this study are available within the supporting information of this article.
