# Supplementary material for: Diel-driven variations of leaf dark respiration and metabolite levels in C3 and C4 grasses
Source: Plant Physiol. 2026 Apr 13;201(2):kiag200. doi: 10.1093/plphys/kiag200 (PMC13316934; doi:10.1093/plphys/kiag200)
Supplement: kiag200_Supplementary_Data [file kiag200_supplementary_data.zip › Supplemtary_Data_PLPHYS-2025-1747_R1.pdf]

MSID: PLPHYS-2025-1747R1

TITLE: Diel-driven variations of leaf dark respiration and metabolite levels in C_3 and C_4 grasses

AUTHORS: Yuzhen Fan; Guillaume Tcherkez; Andrew P. Scafaro; Nicolas L. Taylor; Robert T. Furbank; Susanne von Caemmerer; Owen K. Atkin

SUPPLEMENTARY DATA

Notes S1. The effect of high sucrose concentrations on sucrose transport

A large sucrose pool in maize may facilitate the intracellular transport of sucrose from mesophyll cells to the phloem/sink tissue. In the light, sucrose is synthesised in mesophyll cells in maize (Lunn & Furbank, 1997), which presents a transport challenge, as sucrose must move through bundle sheath cells before reaching the phloem. Given that the intracellular transport of sucrose from mesophyll to bundle sheath cells occurs via diffusion through plasmodesmata or passive transporters (Chen *et al.*, 2021), our results suggest the possibility that maintaining a high sucrose concentration gradient may facilitate diffusion and enhance the efficiency of sucrose transport towards the phloem. Once sucrose reaches the bundle sheath cells, it is thought to be loaded into companion cells via apoplastic phloem loading (Emms *et al.*, 2016; Chen *et al.*, 2021). By contrast, in the dark, sucrose concentrations equilibrate between mesophyll and bundle sheath cells. Starch in bundle sheath cells is degraded and converted to sucrose for export via the same pathway described above.

Notes S2. Starch degradation in C_3 and C_4 plants

One of the aims of this study was to explore changes in starch levels with higher resolution over a diel cycle. Our previous study found that in some C_4 grasses, the starch pool size at midnight was larger than at midday. We argued that this suggests a potentially slower nighttime starch degradation rate than the daytime accumulation rate (Fan *et al.*, 2024). Results reported in this current study support the hypothesis. We found that all examined species accumulated starch through the day, followed by a decline in starch overnight (Fig. 2D). This follows the expected circadian-controlled accumulation during the day and starch depletion at night in C_3 plants (Graf *et al.*, 2010). Interestingly, while starch was nearly exhausted by the end of the night (i.e. 23 h since the day began) in wheat, there was a higher baseline level of starch reserves that were not fully depleted at 23 h since the day began in the three C_4 species (Fig. 2D), similar to previous reports (Czedik-Eysenberg *et al.*, 2016; De Souza *et al.*, 2018). The absence of a complete starch depletion by the end of the night may be due to increased starch production or limited conversion of starch to soluble sugars in source tissue; the latter could be due to low sucrose demand from sink tissue (Stitt & Zeeman, 2012) or negative feedback signalling that

inhibits starch-to-sucrose conversion (Lunn *et al.*, 2014). C_4 leaves have a high potential for starch production because of their greater photosynthesis rates (Weise *et al.*, 2011). However, it remains unclear under what conditions the demands from sink tissues are limited or lower than the starch production in source leaves of C_4 plants. Whether the higher basal levels of starch in C_4 leaves that we measured occur more generally in C_4 species, and what is the cost (e.g. increased starch maintenance respiration), or is there a benefit, to growth and reproductive fitness of an imbalance between leaf source and sink metabolism are unanswered questions that need further exploration.

Figure S1. CO_2-based rate of leaf dark respiration (R_{dark}) and photosynthesis measured at 1,500 µmol quanta m^{-2} s^{-1} light (A_{1500}) of C_3 and C_4 species expressed on three different units at 6 h and 18 h since the day began (equivalent to midday and midnight, respectively: (A) R_{dark} per leaf area; (B) R_{dark} per leaf fresh mass; (C) R_{dark} per leaf dry mass; (D) A_{1500} per leaf area. Data are presented as mean ± SE at each measuring time point. Statistical results of a two-way ANOVA examining time and/or species effect are indicated on Panels (A) to (C). Significant statistical result of a one-way ANOVA examining species effect on A_{1500} is shown in Panel (D). *, P < 0.05; **, P < 0.01; ***, P < 0.001; ns, not significant.

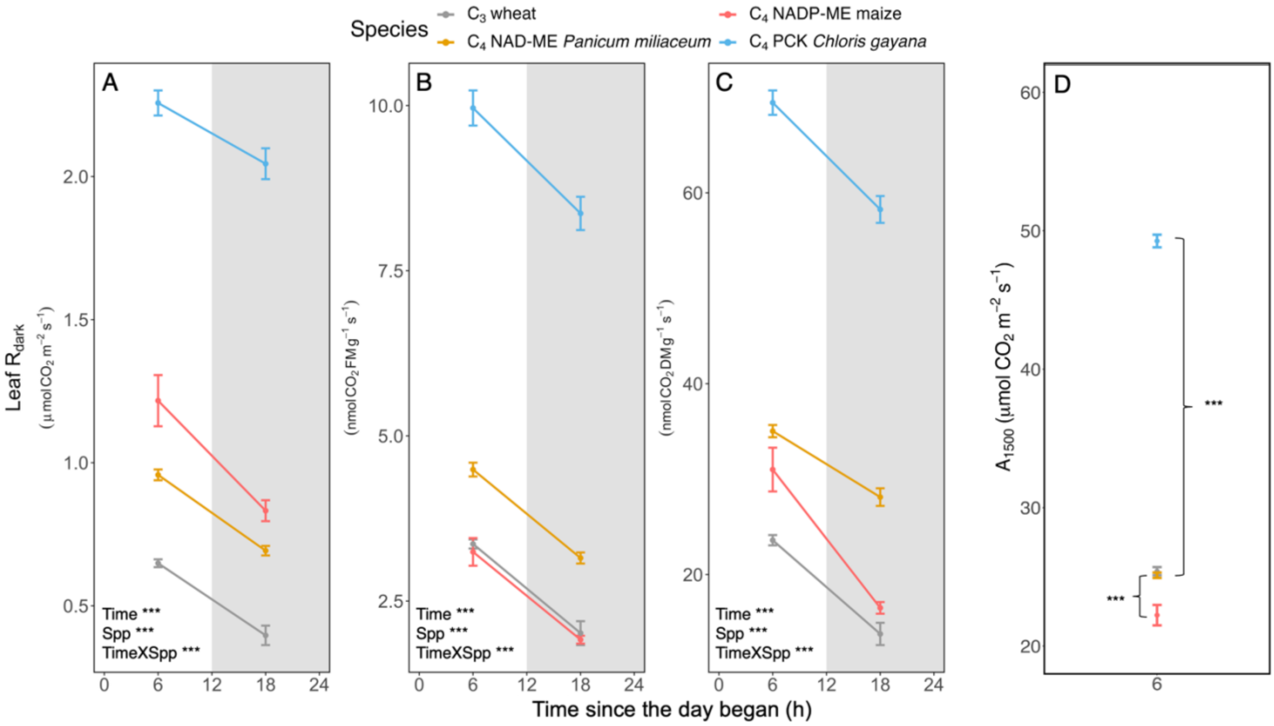

Figure S2. Effect of the diel cycle on relative concentrations of metabolites in C_3 and C_4 leaves associated with primary and secondary metabolic pathways. The x-axis indicates the time since the day began (in hours) when leaves were harvested, and the y-axis denotes the species. Data represent the mean value of multiple replicates (n = 1 - 6) at each time point and are made relative to data collected at the first harvesting time point (3 h since the day began) within each species. As such, relative metabolite concentrations at 3 h since the day began are considered as "1" (indicated as white cells in the first column of every plot), while an increase and a decrease in relative metabolite concentrations in the subsequent hours are indicated as red and blue cells, respectively. The colour gradients of red and blue cells illustrate the degree of changes in relative metabolite concentrations (see legend). Statistical results of the time effect on metabolite levels can be found in Table 1. Pathways associated with C_4 metabolism are indicated with green arrows and overlaid with a green background. BCAA, branched chain amino acids; OPPP, oxidative pentose phosphate pathway; PR, photorespiration; TCAP, mitochondrial tricarboxylic acid pathway. Galactose and aldarate metabolism are also referred to as "sugar interconversions" for simplicity in the main text. Metabolic pathway is structured based on information from Tcherkez *et al.* (2012), Rasmusson *et al.* (2014), Hurtado *et al.* (2017), Fu *et al.* (2020) and Rashid *et al.* (2020).

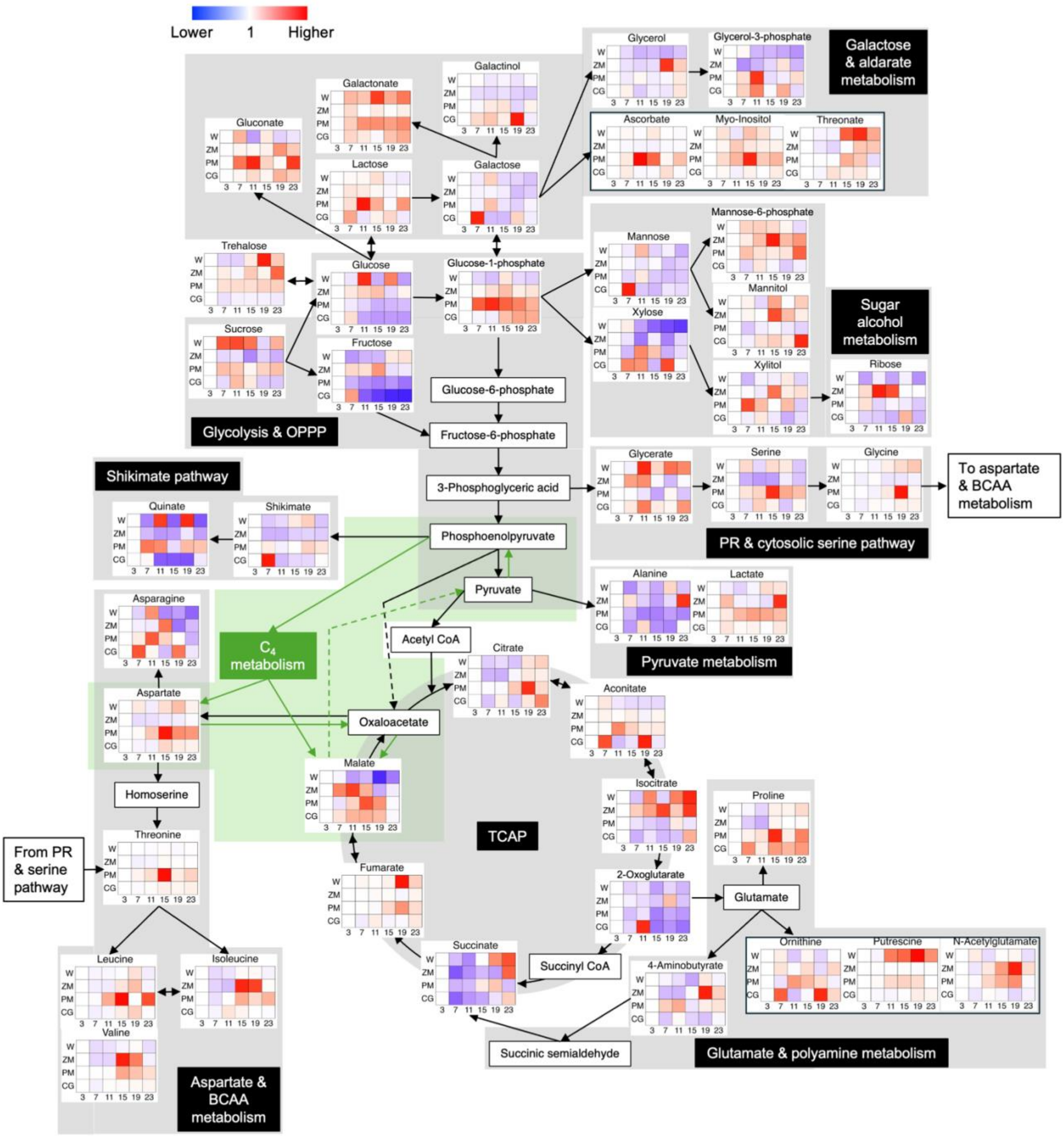

Lower
1
Higher
Galactose & aldarate metabolism
Glycerol
Glycerol-3-phosphate
Galactinol
Galactonate
Gluconate
Lactose
Galactose
Ascorbate
Myo-Inositol
Threonate
Trehalose
Glucose
Glucose-1-phosphate
Sucrose
Fructose
Glucose-6-phosphate
Fructose-6-phosphate
Glycolysis & OPPP
Mannose
Mannose-6-phosphate
Mannitol
Xylose
Xylitol
Ribose
Sugar alcohol metabolism
3-Phosphoglyceric acid
Glycerate
Serine
Glycine
To aspartate & BCAA metabolism
PR & cytosolic serine pathway
Shikimate pathway
Quinate
Shikimate
Phosphoenolpyruvate
Pyruvate
Alanine
Lactate
Pyruvate metabolism
Asparagine
C_4 metabolism
Acetyl CoA
Citrate
Aconitate
Aspartate
Oxaloacetate
Malate
Homoserine
Threonine
From PR & serine pathway
TCAP
Isocitrate
Proline
2-Oxoglutarate
Glutamate
Fumarate
Ornithine
Putrescine
N-Acetylglutamate
Succinate
Succinyl CoA
4-Aminobutyrate
Leucine
Isoleucine
Valine
Succinic semialdehyde
Glutamate & polyamine metabolism
Aspartate & BCAA metabolism
W
ZM
PM
CG
3 7 11 15 19 23

Figure S3. Score plot of multivariate orthogonal partial least squares analysis of metabolites with leaf dark respiration (R_{dark}) expressed per unit of fresh mass as a response variable, coloured by sampling time (hours since the day began).

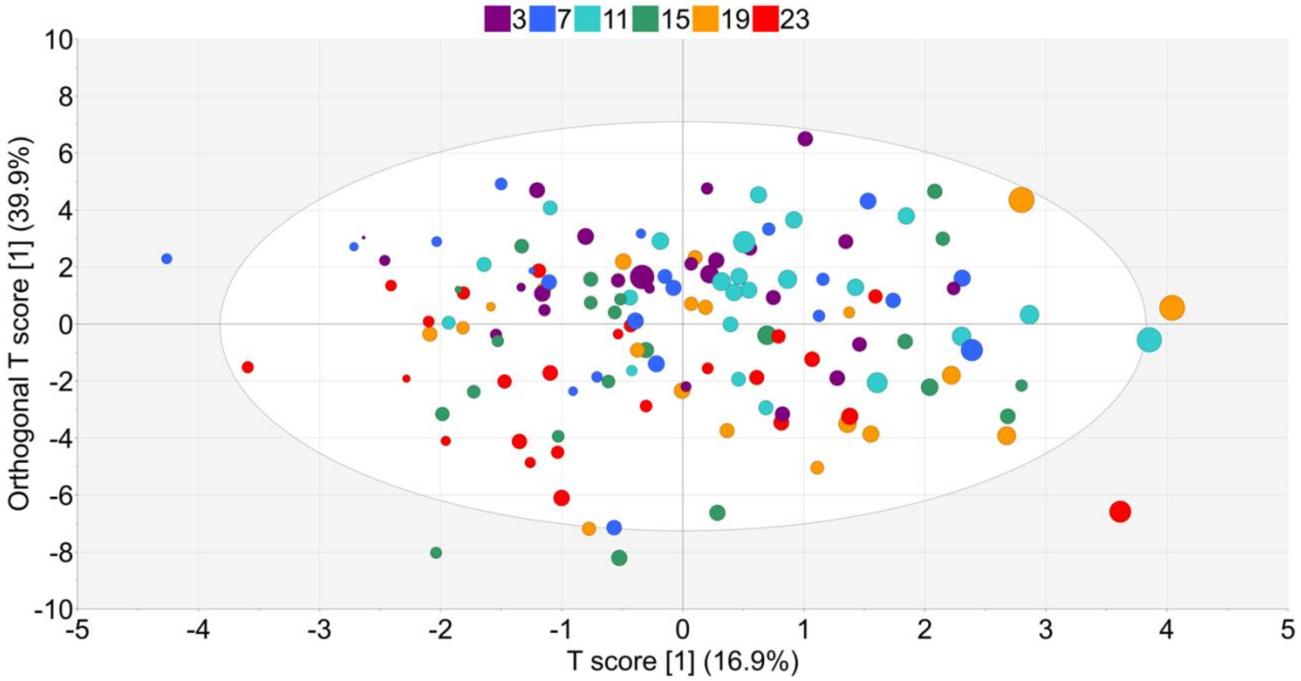

Table S1. Results from a two-way ANOVA test for data shown in Figure 1. Leaf physiological traits were compared between C_3 and C_4 species at six measuring time points. '*', '**' and '***' denotes $P < 0.05$, 0.01 and 0.001, respectively.

	Df	Sum Sq	Mean Sq	F value	*P* value
**Figure 1A – LMA**					
Time	5	156.4	31.3	3.125	**0.011***
Species	3	1117.1	372.4	37.206	**0.000*****
Time × Species	15	246.5	16.4	1.642	0.074
Residuals	113	1130.9	10.0		
**Figure 1B – N concentration**					
	Df	Sum Sq	Mean Sq	F value	*P* value
Time	5	0.007	0.001	0.070	0.997
Species	3	28.850	9.617	458.074	**0.000*****
Time × Species	15	0.146	0.010	0.464	0.954
Residuals	118	2.477	0.021		
**Figure 1C – Total soluble sugar content**					
	Df	Sum Sq	Mean Sq	F value	*P* value
Time	5	9.53	1.91	1.115	0.357
Species	3	99.02	33.01	19.296	**0.000*****
Time × Species	15	57.63	3.84	2.246	**0.008****
Residuals	113	193.29	1.71		
**Figure 1D – TNC**					
	Df	Sum Sq	Mean Sq	F value	*P* value
Time	5	44.21	8.84	3.830	**0.003****
Species	3	154.59	51.53	22.323	**0.000*****
Time × Species	15	53.22	3.55	1.537	0.104
Residuals	114	263.16	2.31		

Table S2. Results from a two-way ANOVA test for data shown in Figure 2. Soluble sugar and starch concentrations were compared between C_3 and C_4 species at six measuring timepoints. '**' and '***' denotes $P < 0.01$ and 0.001, respectively.

**Figure 2A – Glucose**					
	Df	Sum Sq	Mean Sq	F value	*P* value
Time	5	0.279	0.0557	1.563	0.176
Species	3	2.352	0.7841	21.995	**0.000*** **
Time × Species	15	0.717	0.0478	1.341	0.190
Residuals	113	4.028	0.0356		
**Figure 2B – Fructose**					
	Df	Sum Sq	Mean Sq	F value	*P* value
Time	5	1.056	0.211	0.995	0.424
Species	3	10.344	3.448	16.238	**0.000*** **
Time × Species	15	4.856	0.324	1.525	0.108
Residuals	111	23.570	0.212		
**Figure 2C – Sucrose**					
	Df	Sum Sq	Mean Sq	F value	*P* value
Time	5	7.23	1.447	3.309	**0.008** **
Species	3	30.31	10.102	23.107	**0.000*** **
Time × Species	15	20.52	1.368	3.129	**0.000*** **
Residuals	112	48.97	0.437		
**Figure 2D – Starch**					
	Df	Sum Sq	Mean Sq	F value	*P* value
Time	5	28.00	5.599	21.141	**0.000*** **
Species	3	53.65	17.885	67.524	**0.000*** **
Time × Species	15	10.37	0.691	2.609	**0.002** **
Residuals	108	28.61	0.265		

Table S3. Results from a two-way ANOVA test for data shown in Figure 3. Rates of leaf dark respiration (R_{dark}) were compared between C_3 and C_4 species at six measuring timepoints. '*', '**' and '***' denotes $P < 0.05$, 0.01 and 0.001, respectively.

**Figure 3A – R_{dark} per leaf area**					
	Df	Sum Sq	Mean Sq	F value	*P* value
Time	5	0.212	0.043	4.561	**0.000***
Species	3	2.431	0.810	86.972	**0.000***
Time × Species	15	0.109	0.007	0.777	0.700
Residuals	113	1.053	0.009		

**Figure 3B – R_{dark} per leaf fresh mass**					
	Df	Sum Sq	Mean Sq	F value	*P* value
Time	5	14.32	2.86	7.856	**0.000***
Species	3	162.38	54.13	148.476	**0.000***
Time × Species	15	10.52	0.70	1.924	0.028
Residuals	110	40.10	0.36		

**Figure 3C – R_{dark} per leaf dry mass**					
	Df	Sum Sq	Mean Sq	F value	*P* value
Time	5	188	37.5	4.778	**0.000***
Species	3	6431	2143.6	272.926	**0.000***
Time × Species	15	675	45.0	5.733	**0.000***
Residuals	113	887	7.9		

**Figure 3D – R_{dark} per leaf N content**					
	Df	Sum Sq	Mean Sq	F value	*P* value
Time	5	0.198	0.040	5.982	**0.000***
Species	3	3.438	1.146	172.713	**0.000***
Time × Species	15	0.190	0.013	1.913	**0.029***
Residuals	114	0.757	0.007		

Table S4. Top 10 positive and negative correlations between metabolite variables and dimensions in Principal component analysis shown in Figure 4.

	Dim 1		Dim 2	
Positive	Pyroglutamate	0.87	Malate	0.81
	N-Acetylglutamate	0.84	Threitol	0.80
	Isoleucine	0.83	Glycerate	0.69
	Aspartate	0.81	Putrescine	0.65
	4-Aminobutyrate	0.73	Xylitol	0.63
	Valine	0.72	Fumarate	0.62
	Succinate	0.72	Isocitrate	0.58
	Serine	0.70	Threonate	0.58
	Glycerol	0.69	Trehalose	0.54
	Fumarate	0.67	Itaconate	0.54
Negative	Mannose	-0.85	Lactose	-0.60
	Galactose	-0.84	Leucine	-0.50
	Glucose	-0.82	Glycerol	-0.45
	Rhamnose	-0.81	Dulcitol	-0.42
	Shikimate	-0.79	Octadecanate	-0.38
	Fructose	-0.73	Threonine	-0.38
	Quinate	-0.73	Palmitate	-0.36
	Methylmalonate	-0.73	Serine	-0.36
	Aconitate	-0.68	Proline	-0.36
	Lactose	-0.67	Phosphorate	-0.35

Table S5. Top 10 positive and negative correlations between metabolite variables and dimensions in principal component analysis of C_3 and C_4 species shown in Figure 5.

**C_3 Wheat (Figure 5A)**				
	Dim 1		Dim 2	
Positive	Mannose	0.89	Glycerol	0.89
	Ribose	0.87	Phosphorate	0.84
	Glucose	0.87	Glycerol-3-phosphate	0.80
	Galactose	0.86	Valine	0.74
	Quinate	0.82	Leucine	0.71
	Malate	0.81	Gluconate	0.65
	Rhamnose	0.81	Pyroglutamate	0.64
	Asparagine	0.80	2-Oxoglutarate	0.62
	Shikimate	0.78	Serine	0.62
	Xylose	0.75	Galactinol	0.58
Negative	Malonate	-0.76	Putrescine	-0.84
	Benzonate	-0.75	Glycerate	-0.70
	Proline	-0.71	Galactonate	-0.67
	Threonine	-0.65	Caffeic acid	-0.63
	Glycine	-0.63	Myo-Inositol	-0.56
	Isoleucine	-0.60	Threitol	-0.55
	Palmitate	-0.60	Isothreonate	-0.52
	Octadecanate	-0.57	Trehalose	-0.51
	Trehalose	-0.47	Ornithine	-0.45
	Lactate	-0.42	Lactate	-0.43
**C_4 NADP-ME *Zea mays* (Figure 5B)**				
	Dim 1		Dim 2	
Positive	Glycerol	0.93	Galactonate	0.65
	Phosphorate	0.93	Threitol	0.65
	Pyroglutamate	0.90	Oxalate	0.63
	N-Acetylglutamate	0.88	Itaconate	0.62
	Aspartate	0.88	Threonate	0.60
	Serine	0.87	Trehalose	0.59
	Glycerol-3-phosphate	0.86	Xylitol	0.53
	4-Aminobutyrate	0.85	Glucose-1-phosphate	0.50
	Leucine	0.85	Xylose	0.49
	Isoleucine	0.81	Aconitate	0.43
Negative	Galactose	-0.88	Galactinol	-0.60
	Glucose	-0.86	Quinate	-0.51
	Rhamnose	-0.84	Sucrose	-0.40
	Methylmalonate	-0.83	Serine	-0.25
	Putrescine	-0.82	Lactose	-0.25
	Mannose	-0.80	Leucine	-0.17
	Shikimate	-0.75	Shikimate	-0.16
	Asparagine	-0.70	Ascorbate	-0.16
	Ornithine	-0.66	Glycerol	-0.15
	Lactose	-0.65	Phosphorate	-0.15
**C_4 NAD-ME *Panicum miliaceum* (Figure 5C)**				
	Dim 1		Dim 2	

Positive	Pyroglutamate	0.84	Ascorbate	0.93
	Aspartate	0.82	Lactose	0.91
	Benzonate	0.82	Dulcitol	0.90
	Isoleucine	0.78	Gluconate	0.79
	Serine	0.77	Caffeic acid	0.67
	Proline	0.75	Myo-Inositol	0.66
	Leucine	0.75	Methylmalonate	0.60
	Valine	0.74	Glucose-1-phosphate	0.58
	N-Acetylglutamate	0.67	Galactonate	0.58
	Malonate	0.64	Threonine	0.50
Negative	Glucose	-0.88	Isocitrate	-0.74
	Mannose	-0.86	Glycerate	-0.63
	Fructose	-0.81	Alanine	-0.62
	Galactose	-0.74	Malate	-0.56
	Quinate	-0.73	Succinate	-0.47
	Rhamnose	-0.70	Oxalate	-0.46
	Aconitate	-0.63	Citrate	-0.31
	Shikimate	-0.62	2-Oxoglutarate	-0.30
	Ribose	-0.59	Glycine	-0.30
	Asparagine	-0.58	Quinate	-0.29

**C_4 PCK *Chloris gayan*a (Figure 5D)**				
	Dim 1		Dim 2	
Positive	Fumarate	0.91	Phosphorate	0.70
	N-Acetylglutamate	0.84	Glycerol	0.70
	4-Aminobutyrate	0.82	Malonate	0.69
	Isoleucine	0.78	Threonine	0.65
	Pyroglutamate	0.76	Aspartate	0.63
	Succinate	0.72	Proline	0.62
	Alanine	0.72	Benzonate	0.61
	Valine	0.72	Isothreonate	0.58
	Itaconate	0.66	Palmitate	0.51
	Isocitrate	0.65	Leucine	0.45
Negative	Galactose	-0.94	2-Hydroxyglutarate	-0.60
	Mannose	-0.93	Fructose	-0.55
	Shikimate	-0.88	2-Oxoglutarate	-0.51
	Lactose	-0.87	Trehalose	-0.50
	Aconitate	-0.85	Glucose	-0.49
	Dulcitol	-0.79	Sucrose	-0.47
	Glucose	-0.70	Asparagine	-0.44
	Ornithine	-0.68	Caffeic acid	-0.44
	Methylmalonate	-0.67	Xylitol	-0.42
	Xylose	-0.67	Threonate	-0.41

Reference

Chen L, Ganguly DR, Shafik SH, Ermakova M, Pogson BJ, Grof CPL, Sharwood RE, Furbank RT. **2021**. Elucidating the role of SWEET13 in phloem loading of the C_4 grass *Setaria viridis*. *The Plant Journal* **109**: 615–632.

Czedik-Eysenberg A, Arrivault S, Lohse MA, Feil R, Krohn N, Encke B, Nunes-Nesi A, Fernie AR, Lunn JE, Sulpice R, *et al.* **2016**. The interplay between carbon availability and growth in different zones of the growing maize leaf. *Plant Physiology* **172**: 943–967.

De Souza AP, Grandis A, Arenque-Musa BC, Buckeridge MS. **2018**. Diurnal variation in gas exchange and nonstructural carbohydrates throughout sugarcane development. *Functional Plant Biology* **45**: 865–876.

Emms DM, Covshoff S, Hibberd JM, Kelly S. **2016**. Independent and parallel evolution of new genes by gene duplication in two origins of C_4 photosynthesis provides new Insight into the mechanism of phloem loading in C_4 species. *Molecular Biology and Evolution* **33**: 1796–1806.

Fan Y, Tcherkez G, Scafaro AP, Taylor NL, Furbank RT, von Caemmerer S, Atkin OK. **2024**. Variation in leaf dark respiration among C_3 and C_4 grasses is associated with use of different substrates. *Plant Physiology* **195**: 1475–1490.

Fu H, Guo R, Shen WY, Li MX, Liu Y, Zhao ML, Wang XX, Liu XY, Wang SY, Shi LX. **2020**. Changes in the metabolome of two soybean genotypes under drought stress. *Russian Journal of Plant Physiology* **67**: 472–481.

Graf A, Schlereth A, Stitt M, Smith AM. **2010**. Circadian control of carbohydrate availability for growth in *Arabidopsis* plants at night. *Proceedings of the National Academy of Sciences* **107**: 9458–9463.

Hurtado C, Parastar H, Matamoros V, Piña B, Tauler R, Bayona JM. **2017**. Linking the morphological and metabolomic response of *Lactuca sativa* L exposed to emerging contaminants using GC × GC-MS and chemometric tools. *Scientific Reports* **7**: 6546.

Lunn JE, Delorge I, Figueroa CM, Van Dijck P, Stitt M. **2014**. Trehalose metabolism in plants. *The Plant Journal* **79**: 544–567.

Lunn JE, Furbank RT. **1997**. Localisation of sucrose-phosphate synthase and starch in leaves of C_4 plants. *Planta* **202**: 106–111.

Rashid FAA, Scafaro AP, Asao S, Fenske R, Dewar RC, Masle J, Taylor NL, Atkin OK. **2020**. Diel- and temperature-driven variation of leaf dark respiration rates and metabolite levels in rice. *New Phytologist* **228**: 56–69.

Rasmusson AG, Møller IM, Browse J. **2014**. Respiration and lipid metabolism. In: Taiz L, Zeiger E, Møller IM, Murphy A, eds. Plant physiology and development. Massachusetts, US: Sinauer Associates, Incorporated, 223–258.

Stitt M, Zeeman SC. **2012**. Starch turnover: pathways, regulation and role in growth. *Current Opinion in Plant Biology* **15**: 282–292.

Tcherkez G, Boex-Fontvieille E, Mahé A, Hodges M. **2012**. Respiratory carbon fluxes in leaves. *Current Opinion in Plant Biology* **15**: 308–314.

Weise SE, van Wijk KJ, Sharkey TD. **2011**. The role of transitory starch in C_3, CAM, and C_4 metabolism and opportunities for engineering leaf starch accumulation. *Journal of Experimental Botany* **62**: 3109–3118.
